# Disease burden of chronic hepatitis B and complications in China from 2006 to 2050: an individual-based modeling study

**DOI:** 10.1186/s12985-020-01393-z

**Published:** 2020-08-28

**Authors:** Yang Zheng, Jie Wu, Cheng Ding, Kaijin Xu, Shigui Yang, Lanjuan Li

**Affiliations:** State Key Laboratory for Diagnosis and Treatment of Infectious Diseases, Collaborative Innovation Center for Diagnosis and Treatment of Infectious Diseases, The First Affiliated Hospital, College of Medicine, Zhejiang University, Hangzhou, 310003 China

**Keywords:** Chronic hepatitis B, Disease burden, Individual-based model, China

## Abstract

**Background:**

Chronic hepatitis B has become a major public health problem in China. An accurate depiction of the disease burden has not yet been thoroughly conducted. We aimed to project the disease burden of chronic hepatitis B virus (HBV) infection and related complications by modeling various scenarios.

**Method:**

An individual-based Markov model was used to predict disease burden from 2006 through 2050. We simulated 5 scenarios with different annual incidences, diagnoses and nucleotide analog (NA) treatment rates as well as treatment eligibility, which included a natural history without diagnosis or NA therapy, a base case, a World Health Organization (WHO)-proposed target case and two ideal cases.

**Result:**

The natural history scenario is projected to have the fewest HBsAg losses (27.59 million) and highest number of HBV-related deaths (27.19 million). With improved diagnosis and treatment rates of NA therapy, ideal cases have fewer HBV-related deaths (14.46–14.77 million) than do WHO-proposed cases (15.13 million) and base cases (16.89 million), but the proportion of HBsAg loss is similar among them. With a reduction in new infections, the prevalence of chronic HBV in 2050 is expected to be a minimum of 27.03–27.49 million under WHO and ideal cases.

**Conclusion:**

Ideal scenarios 1 and 2 contribute to the lowest disease burden of HBV and its complications in the future, in which new infection control is more effective than increasing diagnosis, treatment rate and treatment eligibility. However, considering the large existing chronic HBV infected population and the low HBsAg loss rate of NA therapy, it is still difficult to avert the increasing trend of cumulative cirrhosis, DC, HCC, LT, and HBV-related death in all scenarios. If new high-potency drugs are not developed, the disease burden of chronic HBV will remain high in the future.

## Background

Chronic hepatitis B virus (HBV) infection has always been a major public health issue in China. In the latest nationwide seroepidemiologic survey conducted in 2006, the prevalence rate of HBsAg positivity in the general population was 7.18% [1]. Although an updated survey was reconducted in 2014, it only covered a population aged 1–29 years [2]. Based on these data, approximately 93 million people were chronically infected with HBV. People living with chronic HBV infection can be classified into several categories according to serum markers and liver function [3, 4], including immune-tolerant carriers, inactive carriers, HBeAg-positive hepatitis, and HBeAg-negative hepatitis. In 2006, an estimated 63–73 million inactive carriers made up the largest group [4–6].

A main concern about chronic HBV infection is its long-term complications. Chronic HBV infection carries a risk of developing into primary liver cancer, which ranks as the sixth-most common cancer worldwide [7]. It also leads to 786,000 HBV-related deaths per year, making it the tenth-leading cause of death worldwide [8]. Over their lifetimes, 15–40% of people with chronic HBV infection can develop complications ranging from hepatocellular carcinoma (HCC) to HBV-related death [9].

Considering these potential progressions and adverse outcomes, the World Health Organization (WHO) endorsed its first global health sector strategy on viral hepatitis in 2016 with the goal of eliminating hepatitis B and C by 2030 [10]. There are various ways to achieve this goal, including preventing new infections and treating current cases. For those already infected, a ‘functional cure’ (HBsAg loss) is the only method available. The international guidelines also suggested treatment for those who are eligible as well as continued monitoring of all currently infected individuals [11]. Nucleotide analog (NA) therapy was first introduced in approximately 2000. Since 2005, high-potency NAs with minimal risk and side effects, such as entecavir (ETV), have been widely applied in China as the first-line recommendation [12, 13]. However, there are still several problems with diagnosis and treatment in China and worldwide. The rates of diagnosis and treatment are only 18.6 and 10.8%, respectively, in China and 10 and 5% worldwide. In addition, as few as 3–7% of chronic HBV patients could undergo HBsAg loss with NA therapy [14]. To improve these problems, both the WHO and China have mentioned increasing the diagnosis and treatment rate and reducing new infections in strategies to eliminate viral hepatitis [15]. Additionally, China updated its guidelines for chronic HBV treatment in 2019, advocating the expansion of NA treatment eligibility to treat more patients [16].

Several previous HBV modeling studies have evaluated the epidemiology of chronic HBV infection in China [17–19]. Those studies found that increased screening and treatment were effective in reducing chronic HBV infection, and enhanced vaccination was beneficial in eliminating transmission. However, previous studies have rarely clearly projected diseases complicated by HBV, including cirrhosis, DC, HCC, and liver transplantation. Meanwhile, there is no cohort study in China thus far that could accurately follow up on the long-term outcomes of all HBV-infected populations, which is quite important for the systematic assessment of health consequences and decision-making. Therefore, our study developed an individual-level Markov model aiming to project the disease burden of total chronic HBV infections, cirrhosis, DC, HCC, LT, and HBV-related death from 2006 to 2050 under various scenarios representing different levels of diagnosis, treatment rates, treatment eligibility, and annual new infections.

## Materials and methods

### Overview

We developed an individual-level Markov model to simulate HBV infection outcomes in individuals with chronic HBV infection from 2006 to 2050. The model projects the prevalence of total chronic HBV infection, HBsAg loss population, incidences of progression to cirrhosis, decompensated cirrhosis (DC), HCC, liver transplantation (LT), and HBV-related deaths. Model construction was performed using TreeAge Pro 2011 Suite (TreeAge Software, Williamstown, MA) **(**see Supplementary Figure 1). Data analysis was performed using R version 3.5.3. Figures were drawn with R version 3.5.3 plus Tableau Desktop 2018 (Tableau Software, Inc. Seattle, WA).

### Patient demographics

Our study constructed a simulated chronic HBV-infected population representing a nationwide baseline in 2006 using the results of the national survey of chronic HBV infection epidemiologic study [20, 21] **(**Supplementary Tables 1, 2). New HBV infections after 2006 were added to the simulated population annually based on reports by the Chinese Centers for Disease Control and Prevention, and the age distribution was derived from published data. Since there are no data regarding age structure after 2017, we adopted the same distribution as that adopted in 2017 [22–24] **(**Supplementary Table 3).

### Model schematics

In the natural history scenario, we simplified and organized chronic HBV infection status into the following: three ‘CHB states’, including HBsAg-positive inactive carriers, HBeAg-negative hepatitis, HBeAg-positive hepatitis; four ‘complication states’, including cirrhosis, DC, HCC, and LT; and three absorbing states, including HBsAg losses, background deaths, and HBV-related deaths (Supplementary Figure 2). Each ‘CHB state’, at predefined probability rates, could either progress into cirrhosis and HCC states or undergo HBsAg loss or background death as absorbing states. All of the ‘complication states’ could transfer to each other or undergo HBV-related death (Supplementary Table 4). We also modeled the annual probability of background death caused by non-liver disease, and the background mortality of the age-standardized rate (ASR) was estimated from the National Bureau of Statistics of China [25].

We did not consider immune tolerance as one of the states here, which was common among infants and children, due to less epidemiologic data being available and the largest population comprising chronic HBV infection in adults who were inactive carriers in China [5, 26–28].

We also assumed that those who met the treatment criteria in the Chinese Medical Association and Asian Pacific Association for the Study of the Liver (APASL) guidelines for HBeAg-negative hepatitis, HBeAg-positive hepatitis and cirrhosis states could receive NA therapy and achieve a virological response (defined as undetectable serum HBV DNA during therapy), thereafter progressing at different probability rates (see Supplementary Table 5) [4, 29]. For those who did not achieve a virological response, we assumed that they had the same progression as that observed in the natural history scenario. For DC, HCC, or LT patients, we assumed that the progression rate was relatively constant regardless of NA therapy.

We did not use different strategies for HBV vaccination in our model because it has already been covered universally, with more than 95% coverage at present, and the blocking of vertical transmission is also highly effective, with an incidence of less than 10/100,000 new HBV infections [30, 31]. Meanwhile, the vaccine’s effect has already been shown as the new infection number every year in our model. We simulated only NA therapy, electing not to include interferon therapy due to its finite coverage, numerous side effects and contraindications [13].

### HBV diagnosis, awareness, and treatment

During the simulation, we assumed that newly enrolled individuals were neither diagnosed nor aware at first, and they could become diagnosed with a certain probability. Once diagnosed, patients were assumed to accept their condition and awareness of HBV infection. A certain proportion of diagnosed patients could receive therapy. Newly treated individuals were divided into virological response or non-virological response states, which were permanent until simulation ended.

### Simulation scenarios

Considering the current problems and improvement plans of diagnosis and therapy, we designed five scenarios to simulate different diagnosis rates, treatment rates, treatment eligibility, and new infection numbers. The clinical characteristics of the simulated scenarios are summarized in Table 1, and the simulation parameters are shown in Table 2**.**

**Table 1:** Clinical characteristics of simulated scenarios

Scenarios	Aspects	Clinical intervention
**Natural history**	***Incidence***	Current effort to lower annual new infection
	***Diagnosis***	No diagnosis
	***Treatment***	No treatment
**Base case**	***Incidence***	Current effort to lower annual new infection
	***Diagnosis***	Current diagnosis rate
	***Treatment***	Current treatment rate
**WHO target**	***Incidence***	Extra effort to lower annual new infection (e.g. higher coverage of neonatal vaccination, catch-up vaccination for adults, health counseling)
	***Diagnosis***	Increase diagnosis rate gradually to 90% in 2030
	***Treatment***	Increase treatment rate gradually to 80% in 2030
**Ideal 1**	***Incidence***	Extra effort to lower annual new infection (e.g. higher coverage of neonatal vaccination, catch-up vaccination for adults, health counseling)
(full diagnosis and treatment)	***Diagnosis***	Increase diagnosis rate rapidly to 100% in 2020
	***Treatment***	Increase treatment rate rapidly to 100% in 2020
**Ideal 2**	***Incidence***	Extra effort to lower annual new infection (e.g. higher coverage of neonatal vaccination, catch-up vaccination for adults, health counseling)
(full treatment eligibility)	***Diagnosis***	Increase diagnosis rate gradually to 90% in 2030
	***Treatment***	Increase treatment rate gradually to 80% in 2030
	***Treatment eligibility***	Expand treatment eligibility rapidly to 100% in 2020

Natural history: we simulated there was only current effort to lower annual new infection (current level of vaccination etc.), no diagnosis or treatment. Base-case: we simulated current effort to lower annual new infection, current diagnosis and treatment rate. WHO target: we simulated extra effort to lower annual new infection (e.g. higher coverage of vaccination), gradually increased diagnosis and treatment rate. Ideal 1: we simulated extra effort to lower annual new infection, rapidly increased diagnosis and treatment rate. Ideal 2: we simulated extra effort to lower annual new infection, gradually increased diagnosis and treatment rate, expanded treatment eligibility

**Table 2:** Parameters of simulated scenarios

Scenarios	Assumption	2006–2017	2018–2020	2021–2030	2031–2050
**Natural**	***Incidence***	Historical data	851,659(2017 incidence)	851,659(2017 incidence)	85,159(2017 incidence)
	***Dx%***	No diagnosis	No diagnosis	No diagnosis	No diagnosis
	***Tx%***	No treatment	No treatment	No treatment	No treatment
**Base-case**	***Incidence***	Historical data	851,659(2017 incidence)	851,659(2017 incidence)	851,659(2017 incidence)
	***Dx%***	18.70%	18.70%	18.70%	18.70%
	***Tx%***	10.83%	10.83%	10.83%	10.83%
**WHO target**	***Incidence***	Historical data	555,858 in 2020 (70% of 2015)*	79,408 in 2030 (10% of 2015)*	79,408(2030 incidence)
	***Dx%***	18.70%	30% in 2020*	90% in 2030*	90%(2030 rate)
	***Tx%***	10.83%	10.83%	80% in 2030*	80%(2030 rate)
**Ideal 1** (Dx% = 1; Tx% = 1)	***Incidence***	Historical data	555,858 in 2020 (70% of 2015)*	79,408 in 2030 (10% of 2015)*	79,408(2030 incidence)
	***Dx%***	18.70%	100%	100%	100%
	***Tx%***	10.83%	100%	100%	100%
**Ideal 2**	***Incidence***	Historical data	555,858 in 2020 (70% of 2015)*	79,408 in 2030 (10% of 2015)*	79,408(2030 incidence)
(Eligible% = 1)	***Dx%***	18.70%	30% in 2020*	90% in 2030*	90%(2030 rate)
	***Tx%***	10.83%	10.83%	80% in 2030*	80%(2030 rate)

***Dx%***: diagnosis rate

***Tx%***: treatment rate (treatment/treatment eligible)

***Eligible%***: treatment eligible proportion (patients indicated for treatment/all hepatitis and cirrhosis patients)

*Dx% and Tx% in 2018–2019 and 2021–2029 were estimated through linear regression (Supplementary Table 6)

The five scenarios were as follows: 1) Natural history scenario: No interventions (diagnosis or treatment) were applied; 2) Base case scenario: The current diagnosis and treatment rate simulated were applied from 2004 to 2050; 3) WHO-proposed target scenario: Gradually increased diagnosis and treatment rates and a reduction in the number of new infections were proposed by the WHO, which illustrated an improved diagnosis/treatment rate (Supplementary Tables 3 and 6); 4) Ideal scenario 1—full capacity of diagnosis and treatment: This scenario simulated that all existing HBV-infected patients would be diagnosed, and all treatment-eligible patients could receive and benefit from NA therapy, but annual new infection cases and treatment eligibility were not changed compared with those of the WHO target scenario (Supplementary Table 3); and 5) Ideal case 2—full treatment eligibility: This scenario simulated that all hepatitis and cirrhosis patients were treatment-eligible, but the annual new infection number, diagnosis rate, and treatment rate were unchanged compared with those of the WHO target scenario (Supplementary Tables 3 and 6). In brief, among all the scenarios, the base case scenario represented the current problems of diagnosis and therapy. The WHO targeted scenario and ideal scenario 1 represented a gradually and rapidly increased diagnosis and treatment rate, respectively. Ideal scenario 2 mainly represented expanded treatment eligibility.

### Validation

We validated our model’s results using authoritative public health data sources, which included the following: the annual cirrhosis and DC incidence from the Institute for Health Metrics and Evaluation (IHME) Global Health Data Exchange (GHDx) online database; the annual HCC incidence from WHO CI5plus/IARC 2010–2012, WHO Globocan 2018, Polaris online database; the annual HBV-related death with WHO Globocan 2018 online database; the annual LT incidence from the China Liver Transplant Registry (CLTR) online database [32–36]; the annual number of cirrhosis deaths from published global disease burden studies [37, 38]; and the total chronic HBV infection prevalence from studies published by Chinese hepatology experts for the base case scenario [22]. In addition, in the natural history scenario, we compared our predicted cumulative 10-year probabilities of HBsAg loss and chronic hepatitis with those in published studies on inactive carriers [39] (Supplementary S 1.5).

### Sensitivity analysis

We performed a 1-way sensitivity analysis on the model in base-case parameters by considering uncertainty in all probability rates. We defined each annual transition probability using the upper range and lower range values and then compared the upper or lower values of cirrhosis, DC, HCC, and LT cumulative incidence and cumulative death with base-case values (Supplementary S 1.6).

## Result

### Validation and sensitivity analysis

Our model projected that the total number of chronic HCV infections in 2016 was 86.06 million, which is equal to the number reported in the epidemiologic data. The projected annual incidence of cirrhosis, DC, HCC, LT from 2010 to 2018, and HBV-related deaths in 2018 closely matched the reported values [32–34, 40] (Supplementary Table 7). Finally, our model’s 10-year cumulative incidence rates of HBsAg loss and hepatitis in inactive carriers closely matched those of a published real-world study [39] (Supplementary Table 8).

Sensitivity analysis revealed that 81.75% (327/400) of all predicted upper or lower values of cirrhosis, DC, HCC, LT cumulative incidence and cumulative death fluctuated within 10, and 96% (384/400) fluctuated within 20% (Supplementary Table 9, 10).

### Cumulative incidence of HBsAg loss and HBV-related deaths

**Fig. 1:**
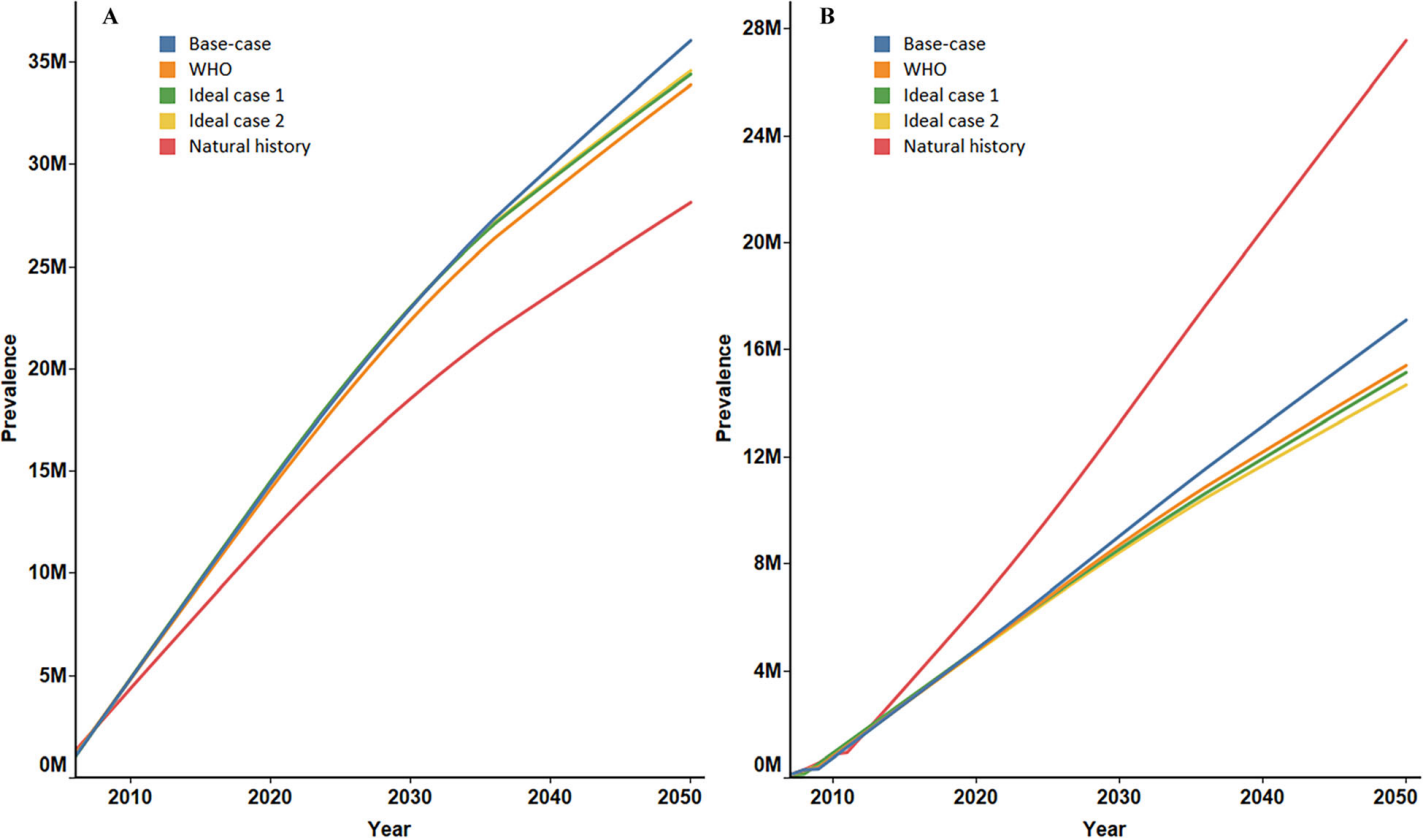
Cumulative incidence of HBsAg loss and HBV-related death. **a**: HBsAg loss number; **b**: HBV-related death number. HBV = hepatitis B virus.

Figure 1a shows the cumulative HBsAg loss in China from 2006 to 2050 under the five scenarios. Under the natural history scenario, HBsAg losses were projected to be 27.59 million, which was the lowest among all scenarios. A total of 32.67 million HBsAg losses were predicted under the WHO target scenario, slightly less than ideal scenario 1 with 33.42 million and ideal scenario 2 with 33.58 million. The highest number of losses occurred under the base case with 35.33 million. The proportions of HBsAg losses in the base case (40.46%), the WHO target scenario (40.01%), ideal scenario 1 (40.71%) and ideal scenario 2 (40.92%) were extremely close, but each exceeded that of the natural history scenario (31.45%) **(**Table 3**).** Figure 1b reveals cumulative HBV-related deaths under different scenarios. Under the natural history scenario, as many as 27.19 million patients were predicted to die of HBV-related reasons by 2050. This number decreased to 16.89 million under the base case and dropped even lower to 15.13 million, 14.77 million, and 14.46 million under scenarios 3–5. Similarly, the proportions of HBV-related deaths in the base case scenario (19.35%), the WHO target scenario (18.53%), ideal scenario 1 (17.99%) and ideal scenario 2 (17.62%) were far lower than that of the natural history scenario (31.00%) **(**Table 3**).**

**Table 3:** Cumulative number and percent of chronic HBV infection, cured, and HBV-related death till 2030 and 2050

Year	Natural		Base-case		WHO		Ideal 1		Ideal 2	
	2030	2050	2030	2050	2030	2050	2030	2050	2030	2050
Total CHB	73.74	43.18	73.53	43.59	68.07	27.49	67.50	27.03	67.51	27.08
Δ CHB	40.14	87.74	40.34	87.32	39.48	81.65	40.06	82.11	40.05	82.07
HBsAg loss	18.79	27.59	23.26	35.33	22.69	32.67	23.42	33.42	23.45	33.58
Percent	46.81%	31.45%	57.65%	40.46%	57.47%	40.01%	58.46%	40.70%	58.55%	40.92%
HBV-RD	13.24	27.19	9.02	16.89	8.77	15.13	8.57	14.77	8.53	14.46
Percent	32.99%	31.00%	22.37%	19.35%	22.21%	18.53%	21.39%	17.99%	21.29%	17.62%
BD	8.11	32.96	8.06	35.10	8.02	33.85	8.07	33.92	8.07	34.03
Percent	20.20%	37.57%	19.98%	40.20%	20.31%	41.46%	20.14%	41.31%	20.15%	41.46%

*Δ CHB* = *basline* + *new infecion* − *ASR* − *HBVrelated death* − *cured*

Reflecting the reduced number of chronic HBV infection

*CHB* Chronic HBV infection, *HBV-RD* HBV-related death, *BD* Background death

### Incidence of chronic HBV complications and death

**Fig. 2:**
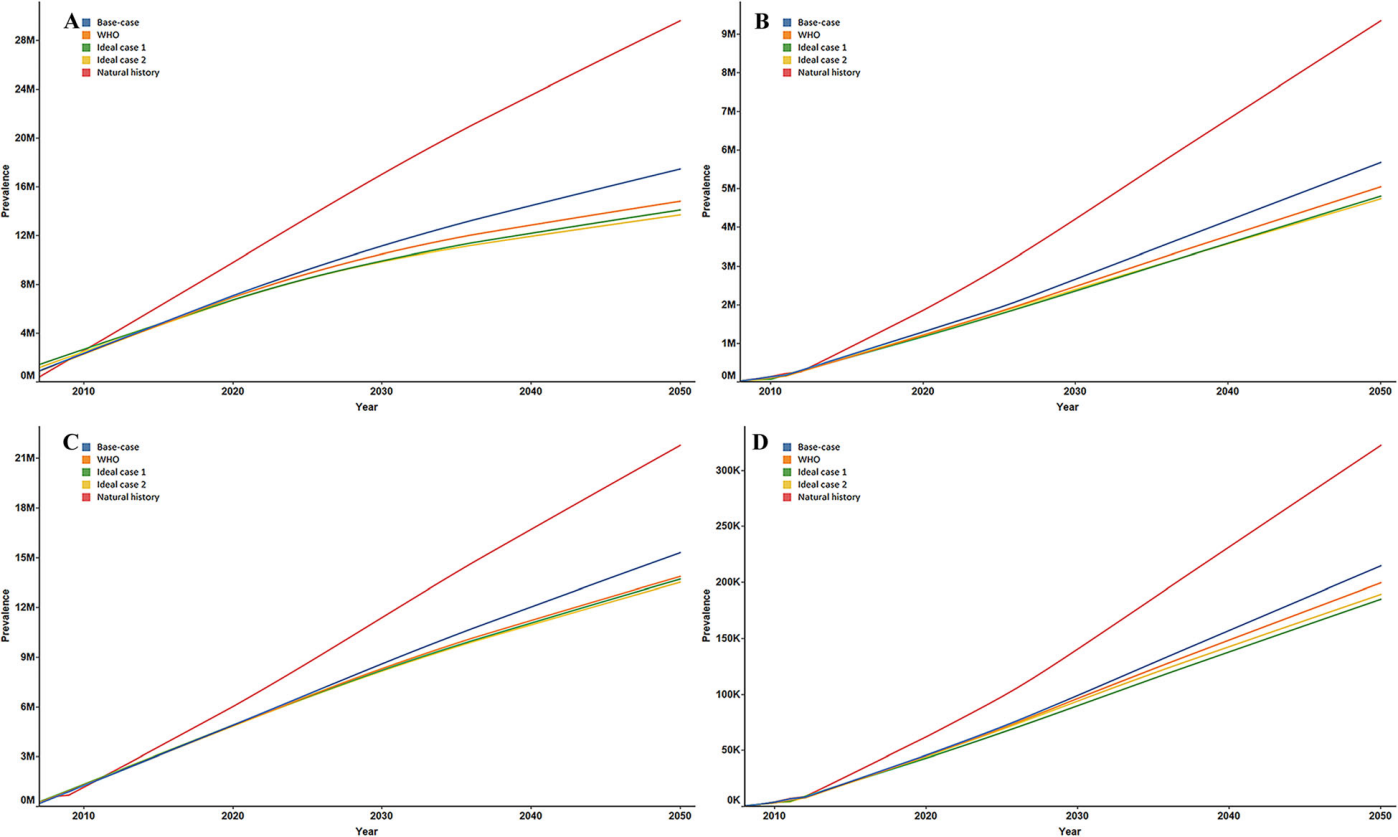
Cumulative incidence of Cirrhosis, DC, HCC, LT. **a**: Cirrhosis incidence; **b**: DC incidence; **c**: HCC incidence; **d**: LT incidence. DC = decompensated cirrhosis; HCC = hepatocellular carcinoma; LT = liver transplantation.

**Fig. 3:**
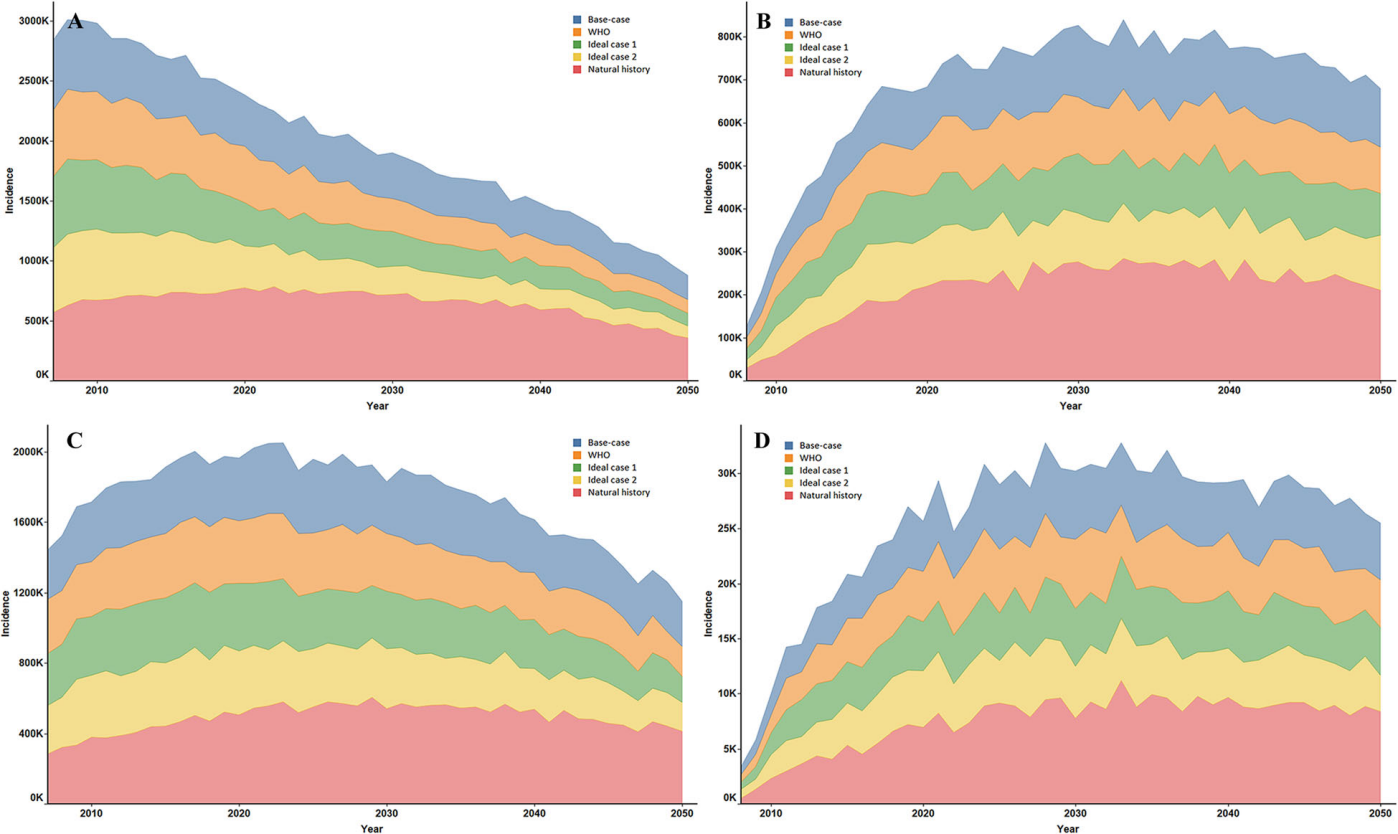
Annual incidence of Cirrhosis, DC, HCC, LT. **a**: Cirrhosis incidence; **b**: DC incidence; **c**: HCC incidence; **d**: LT incidence. DC = decompensated cirrhosis; HCC = hepatocellular carcinoma; LT = liver transplantation.

The cumulative and annual incidence of HBV-related complications and deaths are presented in Fig. 2 and Supplementary Figure 3. We projected that there would be a total of 59.56 million cumulative incidences of HBV-related complications and 27.19 million deaths under the natural history scenario (Supplementary Table 11). Compared to the natural history scenario, the cumulative incidence of HBV-related complications was predicted to decrease significantly to 38.10 million, 33.25 million, 32.02 million, and 31.54 million under scenarios 2–5. Among the four complications, the cumulative incidence of cirrhosis was found to have the largest decline, with 39.87, 49.09, 51.61, and 52.77% reductions under the four non-natural history scenarios. The annual incidence of HBV-related complications and death is presented in Fig. 3 and Supplementary Figure 4. In 2050, HCC death was predicted to be the most common cause of both HBV-related complications and death; the corresponding annual incidence of HCC yielded 411,440, 258,010, 168,840, 147,270, and 165,330, respectively, under the five scenarios.

### Annual incidence of HBeAg negative and positive hepatitis B

**Fig. 4:**
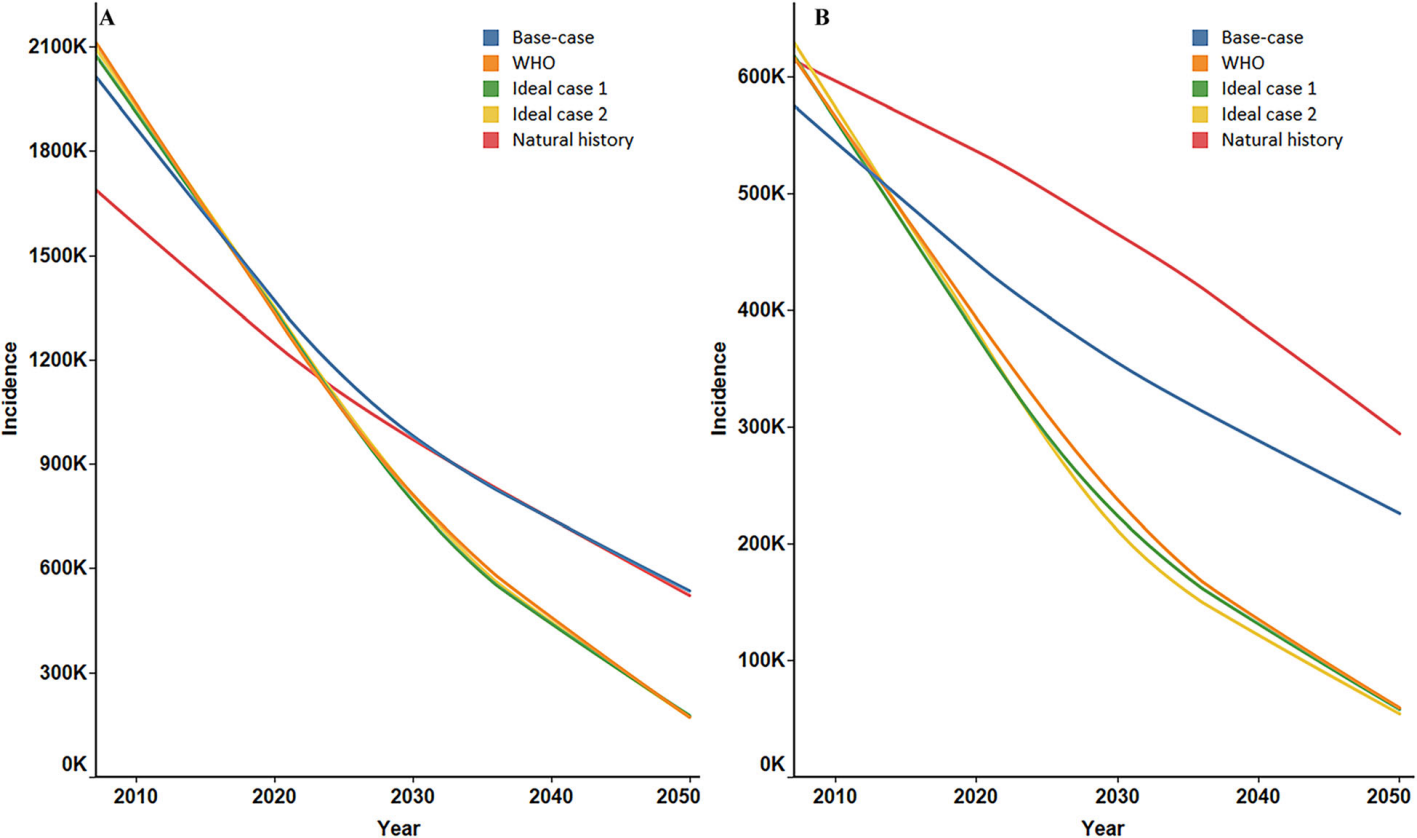
Annual incidence of HBeAg negative and positive hepatitis. **a**: HBeAg-negative hepatitis incidence; **b**: HBeAg-positive hepatitis incidence. HBeAg = hepatitis B e antigen.

Our model also predicted a decreasing trend in the annual incidence of HBeAg-negative and HBeAg-positive hepatitis in Fig. 4. We estimated that the annual incidence of HBeAg-negative hepatitis was the lowest among all the scenarios under the natural history scenario from 2006 (1.69 million) to 2023 (1.13 million). Thereafter, the annual incidences in the natural history and base case scenarios became close and higher than those in the other three scenarios **(**Fig. 4a**)**. The estimated annual incidence of HBeAg-positive hepatitis under the natural history scenario was consistently the highest among all the scenarios; the incidence in 2050 yielded 294,430, which was more than five times that of ideal scenario 2 (54,255) **(**Fig. 4b**)**.

### Prevalence of total chronic HBV infection

The trend of total chronic HBV infection prevalence over time is presented in Supplementary Figure 5. The estimated prevalence was projected to decrease over time under all scenarios, from 93 million in 2006 to 43.18 million, 43.59 million, 27.49 million, 27.03 million, and 27.08 million in 2050 under scenarios 1–5, respectively **(**Table 3**).**

## Discussion

Our model used the most updated domestic data on Chinese hepatitis B epidemiologic studies, national notifiable disease surveillance, published cohort data, and other similar models to systematically and prospectively project chronic HBV infection disease burden in China through 2050. We estimated that the cumulative incidence of cirrhosis, DC, HCC, LT, and HBV-related death would still increase, and the total chronic HBV-infected prevalence would decrease over time under all scenarios. Among them, ideal scenarios 1 and 2 were predicted to have the lowest disease burden of complications and chronic infections. In addition, reducing new infections is projected to be the most effective method now available.

The 100% diagnostic modalities and treatment rates simulated in ideal scenario 1 projected more HBsAg losses, fewer HBV-related deaths and lower total chronic HBV infection prevalence than did gradually increased diagnosis and treatment rates under the WHO-targeted scenario. A simple test-and-treat modality was proven effective in reducing chronic HBV infection and complications. Current HBV screening in China only include childbirth screening and vaccinations, blood product screening, and screening for safe injection practices [41]. More than 80% of HBV infections are unaware of their HBV status [22]. In the United States, screening is recommended for those who are at high risk, including immigrants from places with > 2% prevalence, HIV-infected people, and pregnant women at their first prenatal visits [42, 43]. According to that standard, implementing a 100% HBV screen and achieving full diagnosis in all Chinese individuals is clinically significant due to the medium-high prevalence nationwide. However, given the potentially tremendous economic expense generated by screening and treatment, a further cost-effectiveness analysis is warranted in the future.

Similarly, ideal scenario 2 led to fewer HBV-related deaths and more HBsAg losses than did the WHO-targeted scenario, which proved that increased treatment eligibility was beneficial in reducing the chronic HBV disease burden. In the past, only hepatitis patients with a twofold higher upper limit of normal ALT met the treatment eligibility criteria in China, but recently, the newest Chinese guidelines suggested full treatment for all hepatitis patients with elevated ALT [16]. Our model result provided theoretical evidence and clearly endorsed the recommendation of increasing treatment eligibility by the newest Chinese guidelines.

We also observed a large disparity in the total prevalence between the base case and WHO-targeted scenarios, which were mostly derived from the different settings of annual new infections. Diagnosis and treatment rate only contributed to a minor difference compared with a reduction in new infections. Therefore, controlling new infections is the most effective method available of lowering the chronic HBV disease burden. Although the Chinese government has administered immunization after birth since 1992 and has achieved a significant reduction in total HBV prevalence, there are still 50,000 vertical transmissions and 800,000 new infections every year [1, 22, 44, 45]. New infections were found to be more common in the undeveloped region and among those who were born before 1992 without vaccine protection [46]. Thus, more efforts are still needed, especially on birth immunization in remote regions and catch-up vaccines for susceptible adults.

In addition, the cumulative incidences of cirrhosis, DC, HCC, LT, and HBV-related deaths were predicted to increase over time under all scenarios, implying that NA therapy could only decelerate but not reverse the growing trend. The potential attribution was based on the assumption that only a small group of patients receiving NA therapy would reach a ‘functional cure’ status [47]. Even with the help of Peg-IFN therapy, 3–7% of patients would lose HBsAg, far less than 97% of HCV elimination using DAAs [3, 48, 49]. Additionally, the large baseline of the infected population would generate a large disease burden of hepatitis and complications every year, greatly exceeding the number of individual HBsAg losses. These two reasons explain why DAAs could convert the increasing trend of HCV complications, as shown in our previous modeling study, but not NA therapy in HBV [50]. Hence, we suggest that without new high-potency drugs, it will be difficult to avert the increasing trend of cumulative cirrhosis, DC, HCC, LT and HBV-related deaths.

Our study has several limitations, as a modeling study can never exactly simulate an actual situation, particularly with such complicated disease progression. First, the parameters chosen in the model were selected from different published articles. We selected updated and large-scale population studies of Asians published in high impact factor journals. Probabilities that could not be determined were assumed based on Chinese public data and studies. Second, we paid less attention to the economic points of chronic HBV diagnosis and treatment in the current study; thus, further cost-effectiveness analysis is still warranted given the expense of diagnosis and treatment. Third, data from a national open public health database might be underreported. However, we tried to find unpublished data from expert reviews. Finally, HBV pathogenesis and clinical progression are complicated. Our model simplified and focused on the most important components of these issues.

## Conclusions

In conclusion, this study comprehensively predicts the future HBV burden in China and offers several policy implications. We find that ideal scenario 1 (reduced new infection, rapidly increased diagnosis/treatment rate) and ideal scenario 2 (reduced new infection, rapidly expanded treatment eligibility) contribute to the lowest disease burden of HBV and its complications in the future, in which new infection control is more effective than diagnosis and therapeutic advancements and treatment eligibility expansion. However, considering the high existing chronic HBV infection rate and the low HBsAg loss rate of NA therapy, it is still difficult to avert the increasing trend of cumulative cirrhosis, DC, HCC, LT, and HBV-related death in all simulated scenarios. Hence, if new high-potency drugs are not developed, the disease burden of chronic HBV will remain high in the future.

## Supplementary information

**Additional file 1: Supplementary material 1.** Supplementary materials-A. Model construction methods; B. Additional results. Supplementary materials of the manuscript, including model construction methods and additional results.

## Data Availability

All data generated or analysed during this study are included in this published article and its supplementary information files.

## Abbreviations

WHO: World Health Organization
HBV: Hepatitis B virus
NA: Nucleotide analog
DC: Decompensated cirrhosis
HCC: Hepatocellular carcinoma
DAA: Direct-acting antiviral agent
LT: Liver transplantation
