# Supplementary material for: Disease burden of chronic hepatitis B and complications in China from 2006 to 2050: an individual-based modeling study
Source: Virol J. 2020 Aug 28;17:132. doi: 10.1186/s12985-020-01393-z (PMC7455911; doi:10.1186/s12985-020-01393-z)
Supplement: Supplementary file 1 — Additional file 1: Supplementary material 1. Supplementary materials-A. Model construction methods; B. Additional results. Supplementary materials of the manuscript, including model construction methods and additional results. [file 12985_2020_1393_MOESM1_ESM.docx]

**Supplementary Material A. Model construction methods**

**S 1.1 Model construction**

Model construction and running were utilized by TreeAge Pro 2011 Suite (TreeAge Software, Williamstown, MA). Variables were built to carry numeric values and formula. Trackers were used to record the onset time and cumulative number of events which happened in the progression of simulation. In a single microsimulation trial, a random walk selects a path through the chance nodes in the tree, with higher probability paths being more likely. We used the Markov cycle to restrict the time of simulation. Since the input transition probabilities are annual, each cycle in our model represents one year. Running sample size of microsimulation trial was equal to annual incidence number every year. We simulated the disease progression of chronic HBV-infected patients in China from 2006 to 2050. 2006-2017 period was used as validation with known input parameters, predicted results were collected after 2018.

**
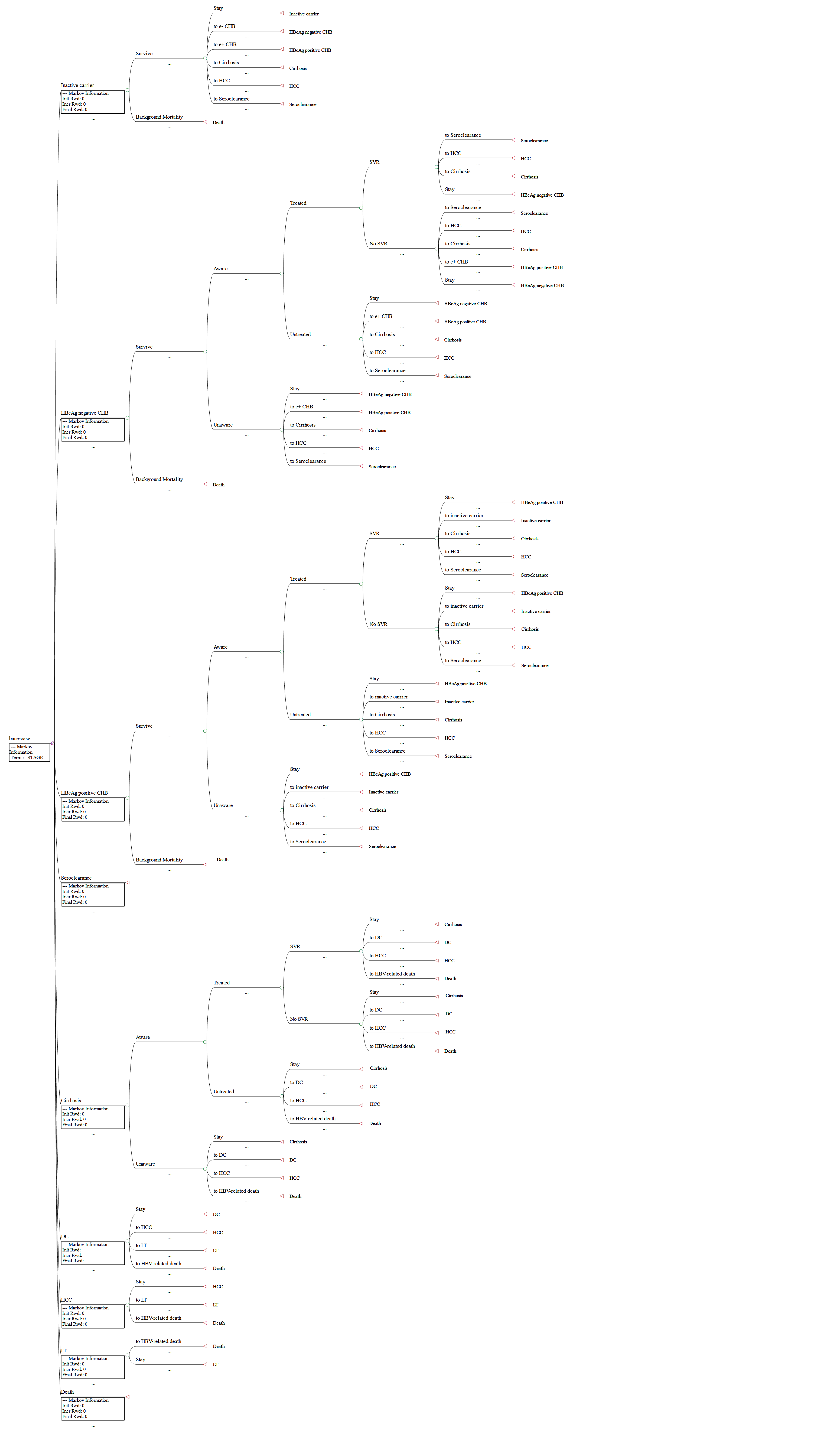
Supplementary Figure 1.Schematic of Markov model in TreeAge Pro software**

**Supplementary Table 1. Estimated annual chronic HBV incidence and age distribution from 2006 to 2050**

| Year | Reported annual HBV incidence | Estimated chronic HBV incidence | Simulation number (proportion) | Age distribution% | | | | | | | | | | | | |
| --- | --- | --- | --- | --- | --- | --- | --- | --- | --- | --- | --- | --- | --- | --- | --- | --- |
|  |  |  |  | 1- | 5- | 10- | 15- | 20- | 25- | 30- | 35- | 40- | 45- | 50- | 55- | 60- |
| 2006 | (prevalence) | 93million | 93000 (0.1%) | 0.74 | 1.17 | 3.46 | 7.51 | 13.16 | 9.48 | 12.20 | 10.53 | 9.84 | 7.09 | 7.69 | 5.88 | 11.25 |
| 2007 | 1169946 | 994454 | 99445 (10%) | 0.59 | 1.01 | 2.20 | 9.58 | 13.24 | 11.47 | 11.49 | 11.56 | 10.26 | 6.44 | 7.31 | 5.29 | 9.56 |
| 2008 | 1169569 | 994134 | 99413 (10%) | 0.54 | 0.78 | 1.70 | 8.16 | 13.36 | 11.25 | 10.89 | 11.53 | 10.44 | 7.44 | 7.48 | 5.87 | 10.55 |
| 2009 | 1179607 | 1002666 | 100266 (10%) | 0.50 | 0.65 | 1.43 | 6.69 | 13.37 | 11.21 | 10.27 | 11.44 | 10.55 | 8.47 | 7.22 | 6.41 | 11.78 |
| 2010 | 1060582 | 901495 | 90149 (10%) | 0.50 | 0.43 | 0.99 | 4.86 | 13.00 | 11.31 | 10.00 | 11.13 | 10.85 | 9.47 | 7.25 | 7.08 | 13.13 |
| 2011 | 1093335 | 929335 | 92933 (10%) | 0.47 | 0.31 | 0.67 | 3.90 | 12.92 | 11.82 | 9.79 | 10.66 | 11.02 | 10.14 | 6.98 | 7.37 | 13.95 |
| 2012 | 1087086 | 924023 | 92402 (10%) | 0.43 | 0.27 | 0.53 | 3.27 | 11.76 | 12.04 | 9.78 | 10.04 | 11.08 | 10.43 | 7.19 | 7.80 | 15.39 |
| 2013 | 962974 | 818528 | 81852 (10%) | 0.41 | 0.25 | 0.44 | 2.86 | 10.40 | 12.07 | 9.81 | 9.68 | 11.23 | 10.56 | 8.00 | 8.01 | 16.27 |
| 2014 | 935702 | 795347 | 79534 (10%) | 0.39 | 0.25 | 0.37 | 2.45 | 9.04 | 12.45 | 9.75 | 9.30 | 11.20 | 10.68 | 8.91 | 7.83 | 17.40 |
| 2015 | 934215 | 794083 | 79408 (10%) | 0.36 | 0.25 | 0.34 | 2.14 | 7.46 | 12.44 | 9.68 | 9.14 | 10.93 | 11.11 | 9.99 | 7.53 | 18.62 |
| 2016 | 942268 | 800928 | 80092 (10%) | 0.31 | 0.23 | 0.30 | 1.86 | 6.36 | 12.39 | 10.11 | 9.04 | 10.53 | 11.31 | 10.97 | 7.17 | 19.43 |
| 2017- | 1001952 | 851659 | 85166 (10%) | 0.31 | 0.23 | 0.30 | 1.86 | 6.36 | 12.39 | 10.11 | 9.04 | 10.53 | 11.31 | 10.97 | 7.17 | 19.43 |

Estimated incidence and age distribution in 2006 derived from nation-wide survey data and population census data in National Bureau of Statistics of China (1, 2); Thereafter annual incidence derived from China CDC (3); However, about 85% of reported incidence were chronic HBV infection according to Hui Zhuang’s (expert in hepatology) article and China CDC unpublished data (4). Age distribution from published epidemiologic study (5).And we assumed incidence and age distribution after 2017 were the same.

**Supplementary Table 2. Population characteristics of chronic HBV infected in 2006**

|  | **Value** | **Range** |
| --- | --- | --- |
| **Total number** | 93 million | 86.39-99.48 |
| **Sex%** |  |  |
| Male | 53.57 % |  |
| Female | 46.43 % |  |
| **Stage distribution** |  |  |
| Inactive carrier | 73 % | 67.7-78.5 |
| HBeAg negative hepatitis | 14.5 % | 11.5-17.5 |
| HBeAg positive hepatitis | 12.5 % | 10.0-15.0 |

**Supplementary Table 3. Estimated annual chronic HBV incidence and age distribution from 2018 to 2050 in WHO target, ideal 1, ideal 2 scenarios**

| Year | Estimated chronic HBV incidence | Simulation number (proportion) | Age distribution% | | Annotation |
| --- | --- | --- | --- | --- | --- |
| 2018 | 651148 | 65115 (10%) | = 2017 |  | |
| 2019 | 603503 | 60350 (10%) |  |  | |
| 2020 | 555858 | 55586 (10%) |  | 30% reduction of 2015 | |
| 2021 | 508213 | 50821 (10%) |  |  | |
| 2022 | 460568 | 46057 (10%) |  |  | |
| 2023 | 412923 | 41292 (10%) |  |  | |
| 2024 | 365278 | 36528 (10%) |  |  | |
| 2025 | 317633 | 31763 (10%) |  |  | |
| 2026 | 269988 | 26999 (10%) |  |  | |
| 2027 | 222343 | 22234 (10%) |  |  | |
| 2028 | 174698 | 17470 (10%) |  |  | |
| 2029 | 127053 | 12705 (10%) |  |  | |
| 2030 | 79408 | 79408 |  | 90% reduction of 2015 | |

2018-2019, 2021-2029: numbers were calculated directly by linear estimation.

Estimated incidence derived from WHO global health sectors strategy on viral hepatitis (6). Age distribution was supposed to be the same with 2017.

**S 1.2 Markov chain diagram of chronic HBV progression**

We used a previously developed Markov model, which had been used to project the disease progression and evaluate long-term outcomes in China, and slightly modified certain transmission possibilities with updated epidemiologic data (7, 8).

Three ‘CHB states’ were defined as following: 1) HBsAg positive inactive carrier defined as HBsAg positive, normal level of ALT ≤ 40 U/L, undetectable or low (＜2,000 IU/mL). 2) HBeAg negative hepatitis defined as HBsAg positive, HBeAg negative, an abnormally elevated level of ALT ＞ 40 U/L. 3) HBeAg positive hepatitis defined as HBsAg positive, HBeAg positive, abnormally elevated level of ALT ＞ 40 U/L.

We didn’t consider immune tolerance as one of states here, which is common among infants and children, due to less epidemiologic data are available and the largest population consist of chronic HBV infection in adults is inactive carriers in China (7, 9-11).

Specific proportion of hepatitis patients (HBeAg negative and/or positive) and cirrhosis states could get NAs therapy

Treatment eligible was consistent with Chinese Medical Association and APASL guidelines (HBV DNA>20 000 IU/ml and ALT > 2×ULN for HBeAg-positive hepatitis, HBV DNA>2 000 IU/ml and ALT > 2×ULN for HBeAg-negative hepatitis, HBV DNA detectable with any ALT for cirrhosis). However, those who could not achieve SVR still went the same disease progression pattern with natural history people. Once patients entered into the end-stage liver diseases, namely DC, HCC, LT, we imaged the progression rate relatively constant regardless of NAs therapy.


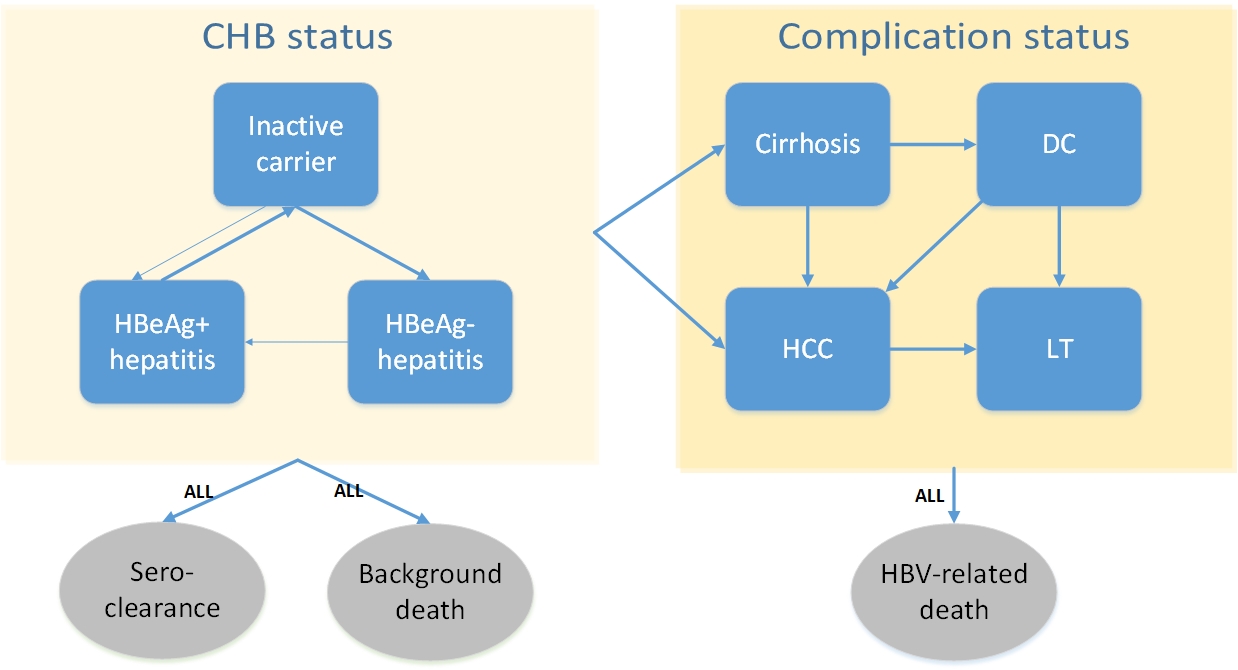


**Supplementary Figure 2. Markov chain diagram**

CHB=chronic hepatitis B infected; DC=decompensated cirrhosis; HCC=hepatic cell carcinoma; LT=liver transplantation

**Supplementary Table 4. Annual transition probabilities**

**of chronic HBV infection in natural history**

| **Transition** | **p** | **Range** | **Reference** |
| --- | --- | --- | --- |
| **from inactive carrier** |  |  |  |
| to spontaneous HBsAg loss | 1.15% | 0.77%-1.83% | (12) |
| to HBeAg-negitive CHB | 1.55% | 0.87-2.78% | (13) |
| to HBeAg-positive CHB | 0.48% | 0.4-1.8% | (14) |
| to cirrhosis | 0.07% | 0-0.2% | (15) |
| to HCC | 0.20% | 0-0.42% | (15) |
| **from HBeAg-negitive CHB** |  |  |  |
| to spontaneous HBsAg loss | 0.75% | 0.675-0.825% | (16) |
| to HBeAg-positive CHB | 1.30% | 1.17-1.43% | (17) |
| to cirrhosis | 2.80% | 1.34-4.3% | (15) |
| to HCC | 0.60% | 0.53-0.72% | (15) |
| **from HBeAg-positive CHB** |  |  |  |
| to spontaneous HBsAg loss | 0.36% | 0.324-0.396% | (16) |
| to inactive carrier | 5% | 4.08%-5.37% | (18) |
| to cirrhosis | 1.60% | 1.3-1.9% | (15) |
| to HCC | 0.60% | 0.53-0.72% | (15) |
| **from cirrhosis** |  |  |  |
| to DC | 4% | 2-5% | (19) |
| to HCC | 3.70% | 3.2-4.14% | (15) |
| to death | 2.57% | 1.45-3.7% | (20) |
| **from DC** |  |  |  |
| to HCC | 8.00% | 8%-9.14% | (19) |
| to LT^*^ | 10% | 5-15% | (19) |
| to death | 7.46% | 6.714-8.206% | (19) |
| **from HCC** |  |  |  |
| to LT^*^ | 4% | 2-6% | Assume |
| to death | 34% | 32.51%-34.89% | (21) |
| **from LT** |  |  |  |
| to death | 4% (1st year) | 3-5% | (22) |
|  | 7.59% (after) | 6.36-8.82% | (22) |

LT^*^: No enough clinical trial data available and extremely unpredictable in the whole country. Figures in the table were cited from a hepatitis B model study performed in Shanghai, China (7).

Since dramatically unbalanced medical and economic development across the whole country, further adjustment according to approved LT centers distribution (23), economic level and population of each province, previous LT statistical reviews and reports were necessary to accurately approach to the real figure (24, 25). We hereby assume the probability of receiving LT was same as Shanghai’s condition, however, only limited population was able to get access to or afford LT. The proportion was estimated to be 6%.

**Supplementary Table 5. Annual transition probabilities**

**of chronic HBV infection related to treatment**

| **Transition** | **p** | **Range** | **Refer** |
| --- | --- | --- | --- |
| **virological response proportion** | 90.50% | 90-91% | (26, 27) |
| **from HBeAg-negative CHB** |  |  |  |
| to HBsAg loss | 1.40% | 1.2-1.5% | (7, 8) |
| to cirrhosis | 0.60% | 0.3-1.2% | (7) |
| to HCC | 0.47% | 0.33-0.6% | (20) |
| **from HBeAg-positive CHB** |  |  |  |
| To HBsAg loss | 4.50% | 4-5% | (26, 27) |
| to inactive carrier | 23.50% | 22-25% | (26, 27) |
| to cirrhosis | 0.20% | 0.1-0.5% | (7, 8) |
| to HCC | 0.47% | 0.33-0.6% | (20) |
| **from cirrhosis** |  |  |  |
| to DC | 2.18% | 1.11%-3.27% | (20) |
| to HCC | 1.06% | 0.954-1.166% | (28) |
| to death | 0.40% | 0.1%-0.71% | (20) |

**S 1.3 Simulation scenarios**

**Background and reasonability of simulated scenarios**

Ideal 1 scenario setting rationale: This scenario simulated the full capacity that the Chinese government could theoretically achieve at the policy level and an upper limit of benefits from 2018 to 2050. This scenario sought to evaluate the impact of improving diagnosis and treatment rates. It also aimed to shed additional light on why the WHO set its target at a 30%-90% level instead of devoting more resources to reaching full capacity.

Ideal 2 scenario setting rationale: All current guidelines listed discrete treatment eligible criteria considering the potentially undesirable extrahepatic adverse events elicited by long-term drug therapy and the considerable economic burden that currently impedes all chronic HBV patients from receiving treatment (29, 30). However, treatment eligibility is always changing. For example, in 2015 the WHO changed its recommendation on initiation of anti-retroviral therapy for HIV from individuals with CD4+ cell counts less than 50 to treatment regardless of CD4+ cell count (31, 32). Therefore, it was reasonable to set a similar full treatment eligibility rate for the chronic hepatitis population to assess the benefits. We assumed that all hepatitis and cirrhosis patients were treatment-eligible. Cases of new infection, diagnosis rate and treatment rate after 2018 were kept the same as in the WHO target scenario (**Supplementary Table 3 and 6)**. Given the lack of clinical data in non-hepatitis patients, we maintained the assumption that this population was not able to receive treatment.

**Mathematical process and illustration of the parameter**

In our model, directly put in parameters were **diagnosis (or awareness) rate** and **treatment rate under the condition of diagnosed**.

However, relevant figures in published epidemiologic study and WHO target were listed as “**diagnosis rate**, **treatment/treatment eligible**”. Treatment-eligible means patients whose clinical status and lab test results meet the criteria of starting antivirus treatment, (For details, please refer to each guideline), and in 2006 this number was 32315 thousand, 37.57% of total chronic HBV population 86000 thousand.

In order to get **treatment rate under the condition of diagnosed**, mathematical process was needed.

*>Mathematical process:*

We defined event diagnose as *Dx*, event treatment as *Tx*.

We further defined that:

Diagnosis rate($Dx\%$)

$$Dx\%=\frac{Dx number}{total number}=d$$

Treatment/treatment eligible ($Tx\%$):

$$Tx\%=\frac{Tx number}{Tx eligible number}=t$$

Eligible proportion ($eligible\%$):

$$eligible\%=\frac{Tx eligible number}{total number}=e$$

Total number as $T$.

So that, probability of getting diagnosed was $p(Dx)=Dx\%=d$, probability of getting treatment was $p\left( Tx \right)=\frac{Tx number}{total number}=\frac{teT}{T}=te$.

We assumed that only those who had already got diagnosed could receive treatment, namely

*Tx* ⊆ *Dx.*

So, the probability of getting **treatment rate under the condition of diagnosed** was

$$p\left( Tx|Dx \right)=\frac{p(TxDx)}{p(Dx)}=\frac{p(Tx)}{p(Dx)}=\frac{te}{d}$$

**Supplementary Table 6. Parameters of WHO target and ideal 2 scenarios**

| Scenarios | WHO target | | | Ideal 2 | | |
| --- | --- | --- | --- | --- | --- | --- |
| Year | Dx% | Tx% | Tx\|Dx% | Dx% | Tx% | Tx\|Dx% |
| 2017 | 18.70% | 10.83% | 21.76% | 18.70% | 10.83% | 21.76% |
| 2018 | 22.47% | 10.83% | 18.11% | 22.47% | 10.83% | 48.20% |
| 2019 | 26.23% | 10.83% | 15.51% | 26.23% | 10.83% | 41.28% |
| 2020 | 30.00% | 10.83% | 13.56% | 30.00% | 10.83% | 36.10% |
| 2021 | 36.00% | 17.75% | 18.52% | 36.00% | 17.75% | 49.30% |
| 2022 | 42.00% | 24.66% | 22.06% | 42.00% | 24.66% | 58.72% |
| 2023 | 48.00% | 31.58% | 24.72% | 48.00% | 31.58% | 65.79% |
| 2024 | 54.00% | 38.50% | 26.78% | 54.00% | 38.50% | 71.29% |
| 2025 | 60.00% | 45.42% | 28.44% | 60.00% | 45.42% | 75.69% |
| 2026 | 66.00% | 52.33% | 29.79% | 66.00% | 52.33% | 79.29% |
| 2027 | 72.00% | 59.25% | 30.92% | 72.00% | 59.25% | 82.29% |
| 2028 | 78.00% | 66.17% | 31.87% | 78.00% | 66.17% | 84.83% |
| 2029 | 84.00% | 73.08% | 32.69% | 84.00% | 73.08% | 87.00% |
| 2030 | 90.00% | 80.00% | 33.40% | 90.00% | 80.00% | 88.89% |

Figures in the years of 2017, 2020, 2030 were cited from published articles or WHO official data.

In the year of 2018-2019, 2021-2029: Dx% and Tx% figures were calculated directly by linear estimation.

Tx|Dx% figures were calculated through the formula.

**S 1.4 Data used for figures plotting**

All the figures in the present study were drawn in scatterplots with x axis of year, y axis of cumulative/annual incidence number, which was processed using locally weighted scatterplot smoothing (LOWESS) regression method rather than raw number.

Locally weighted regression/locally weighted scatterplot smoothing (LOWESS) is a regression method to smooth time series plots in a weighted least-squares fashion to better see relationships between variables and foresee trends, also it could be used to guards against deviant points distorting the smoothed points. The advantage of LOWESS over other regression methods is that LOWESS is a non-parametric strategy, which means the analyzed data does not need to fit some types of distribution (e.g. normal distribution). In addition, LOWESS is very flexible, making it ideal for modelling complex processes for which no theoretical models exist (33-35).

Original figures of cumulative incidence or death were observed with small deviation, default value of frac=0.6667 were used. Original figures of annual incidence or death had a wide fluctuation, for a better fit, we chose frac=0.2 in new graphs plotting (frac tends to zero means overfitting; frac=1 means under-fitting).

**S 1.5 Validation**

We validated our model’s results with authorized public health data including annual cirrhosis and DC incidence number from Institute for Health Metrics and Evaluation (IHME) Global Health Data Exchange (GHDx) online database; annual HCC incidence number from WHO CI5plus/IARC 2010-2012, WHO Globocan 2018, Polaris; annual HBV-related death with WHO Globocan 2018 online database; annual LT incidence number from China Liver Transplant Registry (CLTR) online database (36-40). Annual cirrhosis death from published global disease burden studies (41, 42). Total chronic HBV infection prevalence from published studies conducted by Chinese hepatology experts in base-case scenario (4). In addition, we compared the predicted cumulative 10-year probabilities of HBsAg loss and chronic hepatitis with published studies in inactive carriers in natural history scenario.

**Supplementary Table 7. Validation of base-case intermediate model results to published data**

| Year | Variables | Annual cirrhosis and DC | Annual cirrhosis death | Annual HCC | Annual LT | Annual HBV-related death | | Total prevalent chronic HBV |
| --- | --- | --- | --- | --- | --- | --- | --- | --- |
| 2010 | Reported | 652858 | 45741 | 303627 | 2192 |  | |  |
|  | Modeled | 630600 | 34740 | 337030 | 1940 |  | |  |
| 2011 | Reported | 655262 |  | 300887 | 1908 |  | |  |
|  | Modeled | 610510 |  | 339720 | 2861 |  | |  |
| 2012 | Reported | 656830 |  | 295736 | 2055 |  | |  |
|  | Modeled | 591540 |  | 374010 | 2526 |  | |  |
| 2013 | Reported | 656928 |  |  | 2420 |  | |  |
|  | Modeled | 602600 |  |  | 3270 |  | |  |
| 2014 | Reported | 655548 |  |  | 2840 |  | |  |
|  | Modeled | 632890 |  |  | 3955 |  | |  |
| 2015 | Reported | 652159 |  | 372880 | 2680 |  | |  |
|  | Modeled | 579500 |  | 376870 | 4006 |  | |  |
| 2016 | Reported | 646291 |  | 328648 | 3687 |  | | 86 million |
|  | Modeled | 608960 |  | 367640 | 3715 |  | 86.08 million | |
| 2017 | Reported | 637249 | 75159 |  | 4746 |  | |  |
|  | Modeled | 610610 | 65260 |  | 4484 |  | |  |
| 2018 | Published |  |  | 314294 | 6290 | 368960 | |  |
|  | Modeled |  |  | 354560 | 4449 | 423620 | |  |

**Supplementary Table 8. Validation of modeled natural history to published data**

| Initial state | Variables | 10-year cumulative previous study % | Model prediction % | Reference |
| --- | --- | --- | --- | --- |
| inactive carrier | |  |  |  |
|  | HBsAg loss | 8.10 | 10.3 | (43) |
|  | reactive chronic hepatitis B | 17.40 | 16.36 | (43) |

**S 1.6 Sensitivity analysis**

We did a 1-way sensitivity analysis on the model by replacing each annual transition probability and demographic parameter with both upper range and lower range value.

And evaluating the impact on the change of cirrhosis, DC, HCC, LT cumulative incidence and their cumulative death number in comparison with base-case scenario.

**Supplementary Table 9. Results of 1-way sensitivity analysis (Part I)**

|  |  | **Cumulative incidence from 2006 to 2050 (percent difference from base-case)** | | | | | | | |
| --- | --- | --- | --- | --- | --- | --- | --- | --- | --- |
| **parameter** | | **C** | **DC** | **HCC** | **LT** | **C death** | **DC death** | **HCC death** | **LT death** |
| base-case results | | 13.81 | 4.76 | 13.27 | 183.35 | 1.72 | 1.33 | 11.60 | 129.59 |
| **natural-history transition probabilities** | | |  |  |  |  |  |  |  |
| **from inactive carrier** | | |  |  |  |  |  |  |  |
| to HBsAg loss | 0.77% | 14.23(3.05%) | 4.87 (2.39%) | 13.7 (3.21%) | 191.23 (4.3%) | 1.78 (3.56%) | 1.4 (4.92%) | 11.98 (3.28%) | 133.92 (3.34%) |
| to HBsAg loss | 1.83% | 13.23 (-4.15%) | 4.45 (-6.49%) | 12.71 (-4.22%) | 178.36 (-2.72%) | 1.65 (-3.61%) | 1.27 (-4.53%) | 11.19 (-3.55%) | 124.97 (-3.56%) |
| to HBeAg- CHB | 0.87% | 12.16 (-11.89%) | 4.14 (-12.99%) | 12.54 (-5.54%) | 167.37 (-8.72%) | 1.44 (-16.28%) | 1.18 (-11.48%) | 10.94 (-5.65%) | 118.53 (-8.54%) |
| to HBeAg- CHB | 2.78% | 16.4 (18.79%) | 5.57 (17.16%) | 14.62 (10.15%) | 212.45 (15.87%) | 2.05 (19.38%) | 1.51 (13.73%) | 12.73 (9.78%) | 149.62 (15.45%) |
| to HBeAg+ CHB | 0.40% | 12.86 (-6.85%) | 4.31 (-9.44%) | 13.13 (-1.11%) | 167.26 (-8.77%) | 1.61 (-6.31%) | 1.07 (-19.29%) | 11.64 (0.34%) | 117.19 (-9.57%) |
| to HBeAg+ CHB | 1.80% | 15.35 (11.17%) | 5.32 (11.84%) | 14.21 (7.04%) | 203.2 (10.83%) | 2.07 (20.95%) | 1.46 (9.92%) | 12.33 (6.36%) | 138.54 (6.91%) |
| to cirrhosis | 0.20% | 16.54 (19.82%) | 5.49 (15.4%) | 14.16 (6.67%) | 219.4 (19.67%) | 2.04 (18.93%) | 1.46 (9.67%) | 12.13 (4.61%) | 145.7 (12.43%) |
| to HCC | 0.42% | 13.89 (0.61%) | 4.7 (-1.24%) | 16.61 (25.17%) | 212.52 (15.91%) | 1.89 (10.02%) | 1.37 (3.2%) | 14.64 (26.25%) | 159.53 (23.1%) |
| **from HBeAg-negitive CHB** | |  |  |  |  |  |  |  |  |
| to HBsAg loss | 0.68% | 13.55 (-1.89%) | 4.7 (-1.17%) | 13.36 (0.66%) | 187.1 (2.05%) | 1.68 (-2.19%) | 1.34 (0.88%) | 11.49 (-0.89%) | 133.96 (3.37%) |
| To HBsAg loss | 0.83% | 13.83 (0.2%) | 4.82 (1.37%) | 13.14 (-1.04%) | 191.85 (4.64%) | 1.79 (4.64%) | 1.27 (-4.64%) | 11.41 (-1.64%) | 134.88 (4.08%) |
| to HBeAg+ CHB | 1.17% | 14.12 (2.25%) | 4.9 (3.08%) | 13.65 (2.83%) | 188.69 (2.92%) | 1.72 (0.05%) | 1.47 (10.4%) | 11.84 (2.1%) | 140.38 (8.32%) |
| to HBeAg+ CHB | 1.43% | 13.82 (0.09%) | 4.57 (-3.85%) | 13.35 (0.59%) | 192.38 (4.92%) | 1.56 (-8.91%) | 1.26 (-5.47%) | 11.58 (-0.13%) | 137.45 (6.06%) |
| to cirrhosis | 1.34% | 11.05 (-19.96%) | 3.29 (-30.85%) | 12.85 (-3.17%) | 155.62 (-15.12%) | 1.29 (-24.78%) | 0.77 (-42.06%) | 11.16 (-3.75%) | 110.46 (-14.76%) |
| to cirrhosis | 4.30% | 16.72 (21.13%) | 5.67 (19.33%) | 14.29 (7.64%) | 204.3 (11.43%) | 2.28 (32.97%) | 1.61 (21.13%) | 12.54 (8.17%) | 147.46 (13.79%) |
| to HCC | 0.53% | 13.63 (-1.27%) | 4.38 (-7.95%) | 13.63 (2.69%) | 171.87 (-6.26%) | 1.7 (-0.67%) | 1.21 (-8.97%) | 11.94 (2.93%) | 121.22 (-6.46%) |
| to HCC | 0.72% | 13.59 (-1.6%) | 4.85 (1.98%) | 12.96 (-2.37%) | 184.59 (0.68%) | 1.68 (-2.23%) | 1.41 (5.84%) | 11.22 (-3.22%) | 135.48 (4.54%) |
| **from HBeAg-positive CHB** | |  |  |  |  |  |  |  |  |
| to HBsAg loss | 0.32% | 14.19 (2.77%) | 4.7 (-1.11%) | 12.85 (-3.22%) | 186.38 (1.65%) | 1.63 (-4.92%) | 1.32 (-0.7%) | 11.1 (-4.25%) | 138.71 (7.04%) |
| to HBsAg loss | 0.40% | 14 (1.37%) | 4.69 (-1.46%) | 13.06 (-1.63%) | 178.54 (-2.62%) | 1.68 (-1.87%) | 1.4 (4.87%) | 11.46 (-1.2%) | 127.14 (-1.89%) |
| **Supplementary Table 9. Cont’d** | | | | | | | | | |
|  |  | **Cumulative incidence from 2006 to 2050 (percent difference from base-case)** | | | | | | | |
| **parameter** | **C** | **DC** | **HCC** | **LT** | **C death** | **DC death** | **HCC death** | **LT death** | **parameter** |
| base-case results | 13.81 | 4.76 | 13.27 | 183.35 | 1.72 | 1.33 | 11.60 | 129.59 | base-case results |
| to inactive carrier | 4.08% | 13.41 (-2.85%) | 4.64 (-2.52%) | 13.47 (1.45%) | 178.11 (-2.86%) | 1.79 (4.62%) | 1.28 (-4.05%) | 11.85 (2.22%) | 131.57 (1.53%) |
| to inactive carrier | 5.37% | 13.92 (0.84%) | 4.87 (2.36%) | 14.01 (5.53%) | 180.97 (-1.3%) | 1.76 (2.78%) | 1.47 (10.09%) | 12.39 (6.83%) | 121.43 (-6.3%) |
| to cirrhosis | 1.30% | 13.32 (-3.51%) | 4.41 (-7.16%) | 13.86 (4.43%) | 194.01 (5.82%) | 1.62 (-5.28%) | 1.07 (-19.93%) | 11.97 (3.26%) | 138.85 (7.15%) |
| to cirrhosis | 1.90% | 14.21 (2.93%) | 4.97 (4.61%) | 13.63 (2.68%) | 191.39 (4.39%) | 1.79 (4.43%) | 1.31 (-1.36%) | 11.74 (1.23%) | 125.77 (-2.95%) |
| to HCC | 0.53% | 13.69 (-0.82%) | 4.62 (-2.88%) | 13.49 (1.6%) | 178.1 (-2.86%) | 1.61 (-6.31%) | 1.24 (-6.53%) | 11.88 (2.43%) | 126.47 (-2.41%) |
| to HCC | 0.72% | 14.23 (3.07%) | 4.75 (-0.2%) | 13.81 (4.04%) | 196.02 (6.91%) | 1.69 (-1.49%) | 1.43 (7.48%) | 12.07 (4.09%) | 139.7 (7.8%) |
| **from cirrhosis** |  |  |  |  |  |  |  |  |  |
| to DC | 2% | 14.44 (4.58%) | 3.87 (-18.57%) | 13.65 (2.81%) | 152.15 (-17.01%) | 2.07 (20.72%) | 1.2 (-9.79%) | 12.08 (4.17%) | 104.24 (-19.56%) |
| to DC | 5% | 13.77 (-0.28%) | 4.88 (2.64%) | 13.24 (-0.22%) | 194.45 (6.05%) | 1.62 (-5.26%) | 1.36 (2.22%) | 11.36 (-2.01%) | 152.4 (17.6%) |
| to HCC | 3.20% | 13.92 (0.85%) | 4.81 (1.07%) | 14.02 (5.59%) | 197.55 (7.75%) | 1.68 (-2.15%) | 1.21 (-9.06%) | 12.26 (5.73%) | 139.42 (7.59%) |
| to HCC | 4.14% | 14.46 (4.72%) | 4.91 (3.25%) | 12.87 (-3.08%) | 178.35 (-2.73%) | 1.91 (11.56%) | 1.3 (-2.7%) | 11.25 (-2.97%) | 133.55 (3.06%) |
| to death | 1.45% | 14.03 (1.65%) | 4.86 (2.25%) | 13.35 (0.59%) | 188.85 (3%) | 1.25 (-26.95%) | 1.42 (6.85%) | 11.75 (1.3%) | 135.44 (4.51%) |
| to death | 3.70% | 13.48 (-2.37%) | 4.45 (-6.42%) | 13.5 (1.7%) | 189.77 (3.5%) | 2 (16.8%) | 1.11 (-16.6%) | 11.68 (0.75%) | 137.44 (6.06%) |
| **from DC** |  |  |  |  |  |  |  |  |  |
| to HCC | 8% | 13.81 (0%) | 4.76 (0%) | 13.27 (0%) | 183.35 (0%) | 1.72 (0%) | 1.33 (0%) | 11.6 (0%) | 129.59 (0%) |
| to HCC | 9.14% | 13.93 (0.91%) | 4.68 (-1.57%) | 13.31 (0.31%) | 177.51 (-3.18%) | 1.65 (-3.97%) | 1.18 (-11.65%) | 11.64 (0.39%) | 122.76 (-5.27%) |
| to death | 6.71% | 13.91 (0.78%) | 4.69 (-1.45%) | 13.41 (1%) | 186.04 (1.47%) | 2.52 (47.17%) | 1.06 (-20.25%) | 11.65 (0.43%) | 124.79 (-3.71%) |
| to death | 8.21% | 13.76 (-0.36%) | 4.51 (-5.21%) | 13.08 (-1.48%) | 177.6 (-3.14%) | 1.89 (10.45%) | 1.27 (-4.5%) | 11.35 (-2.1%) | 130.42 (0.64%) |
| **from HCC** |  |  |  |  |  |  |  |  |  |
| to death | 32.51% | 13.66 (-1.04%) | 4.52 (-4.88%) | 13.1 (-1.27%) | 191.58 (4.49%) | 2.62 (52.91%) | 1.27 (-4.76%) | 11.36 (-2.03%) | 132.81 (2.49%) |
| to death | 34.89% | 13.6 (-1.48%) | 4.67 (-1.86%) | 13.61 (2.56%) | 174.8 (-4.66%) | 1.63 (-5.21%) | 1.26 (-5.05%) | 12.09 (4.24%) | 130.81 (0.94%) |
|  |  |  |  |  |  |  |  |  |  |
| **Supplementary Table 9. Cont’d** | | | | | | | | | |
|  |  | **Cumulative incidence from 2006 to 2050 (percent difference from base-case)** | | | | | | | |
| **parameter** | **C** | **DC** | **HCC** | **LT** | **C death** | **DC death** | **HCC death** | **LT death** | **parameter** |
| base-case results | 13.81 | 4.76 | 13.27 | 183.35 | 1.72 | 1.33 | 11.60 | 129.59 | base-case results |
| **other** |  |  |  |  |  |  |  |  |  |
| SVR proportion | 90.00% | 13.48 (-2.38%) | 4.48 (-5.89%) | 13.5 (1.69%) | 176.46 (-3.76%) | 1.66 (-3.27%) | 1.41 (5.92%) | 11.79 (1.68%) | 132.53 (2.27%) |
| SVR proportion | 91% | 14.13 (2.36%) | 4.69 (-1.38%) | 13.37 (0.69%) | 174.38 (-4.89%) | 1.8 (5.06%) | 1.34 (0.75%) | 11.65 (0.47%) | 127.09 (-1.93%) |
| Dx% | 16.83% | 14.82 (7.34%) | 4.83 (1.55%) | 13.69 (3.13%) | 171.41 (-6.51%) | 2.09 (21.79%) | 1.42 (6.29%) | 11.92 (2.76%) | 121.55 (-6.21%) |
| Dx% | 20.57% | 14.01 (1.5%) | 4.93 (3.63%) | 13.41 (0.99%) | 186.19 (1.55%) | 1.65 (-3.87%) | 1.15 (-13.94%) | 11.89 (2.5%) | 123.92 (-4.38%) |
| Tx/Dx | 19.58% | 13.72 (-0.62%) | 4.85 (2.08%) | 13.64 (2.76%) | 203.48 (10.98%) | 1.66 (-3.05%) | 1.38 (3.31%) | 11.67 (0.63%) | 139.81 (7.89%) |
| Tx/Dx | 23.94% | 13.96 (1.1%) | 4.74 (-0.32%) | 13.9 (4.72%) | 184.66 (0.71%) | 1.68 (-1.87%) | 1.24 (-7.12%) | 12.04 (3.84%) | 131.89 (1.78%) |

C, DC, HCC items’ unit: million

LT items’ unit: thousand

**Supplementary Table 10. Results of 1-way sensitivity analysis (part II)**

|  |  |  |  | **Cumulative incidence from 2006 to 2050 (percent difference from base-case)** | | | | | | | |
| --- | --- | --- | --- | --- | --- | --- | --- | --- | --- | --- | --- |
| **parameter** |  |  |  | **C** | **DC** | **HCC** | **LT** | **C death** | **DC death** | **HCC death** | **LT death** |
| base-case results |  |  |  | 13.81 | 4.76 | 13.27 | 183.35 | 1.72 | 1.33 | 11.60 | 129.59 |
| **chronic HBV-infected population characteristics** | | | | | | | | | | | |
| **total chronic HBV infected population(million)** | | | |  |  |  |  |  |  |  |  |
| in 2006 | 86.39 |  |  | 13.21 (-4.32%) | 4.47 (-5.94%) | 12.58 (-5.19%) | 164.46 (-10.3%) | 1.62 (-5.39%) | 1.34 (0.96%) | 11.15 (-3.82%) | 119.62 (-7.69%) |
| in 2006 | 99.48 |  |  | 14.67 (6.27%) | 4.95 (4.2%) | 13.97 (5.22%) | 176.82 (-3.56%) | 1.93 (12.58%) | 1.55 (16.49%) | 12.28 (5.88%) | 127.67 (-1.48%) |
| **stage distribution of chronic HBV infected population** | | | | |  |  |  |  |  |  |  |
| Inactive carrier | **67.7** | 17.15 | 15.15 | 15.21 (10.14%) | 5.2 (9.29%) | 13.65 (2.81%) | 185.52 (1.18%) | 1.96 (14.03%) | 1.57 (18.02%) | 12.01 (3.57%) | 129.23 (-0.28%) |
| Inactive carrier | **78.5** | 11.75 | 9.75 | 13.51 (-2.17%) | 4.25 (-10.54%) | 12.77 (-3.76%) | 159.51 (-13%) | 1.86 (8.64%) | 1.26 (-5.16%) | 11.15 (-3.82%) | 109.67 (-15.37%) |
| HBeAg- CHB | 74.5 | **11.5** | 14 | 13.52 (-2.07%) | 4.68 (-1.63%) | 13.83 (4.16%) | 173.33 (-5.47%) | 1.61 (-6.22%) | 1.2 (-10.19%) | 12.18 (5.05%) | 126.62 (-2.3%) |
| HBeAg- CHB | 71.5 | **17.5** | 11 | 14.9 (7.94%) | 5.22 (9.87%) | 13.6 (2.46%) | 207.77 (13.32%) | 1.77 (3.27%) | 1.37 (3.12%) | 11.79 (1.67%) | 146.12 (12.76%) |
| HBeAg+ CHB | 74.25 | 15.75 | **10** | 13.78 (-0.22%) | 4.54 (-4.42%) | 13.82 (4.14%) | 170.23 (-7.15%) | 2 (16.47%) | 1.3 (-2.38%) | 12.07 (4.06%) | 121.28 (-6.41%) |
| HBeAg+ CHB | 71.75 | 13.25 | **15** | 14.03 (1.58%) | 4.78 (0.46%) | 13.57 (2.21%) | 202.02 (10.18%) | 1.72 (0.06%) | 1.28 (-3.99%) | 11.65 (0.47%) | 148.29 (14.43%) |

C, DC, HCC items’ unit: million

LT items’ unit: thousand

**Supplementary Material B. Additional results**


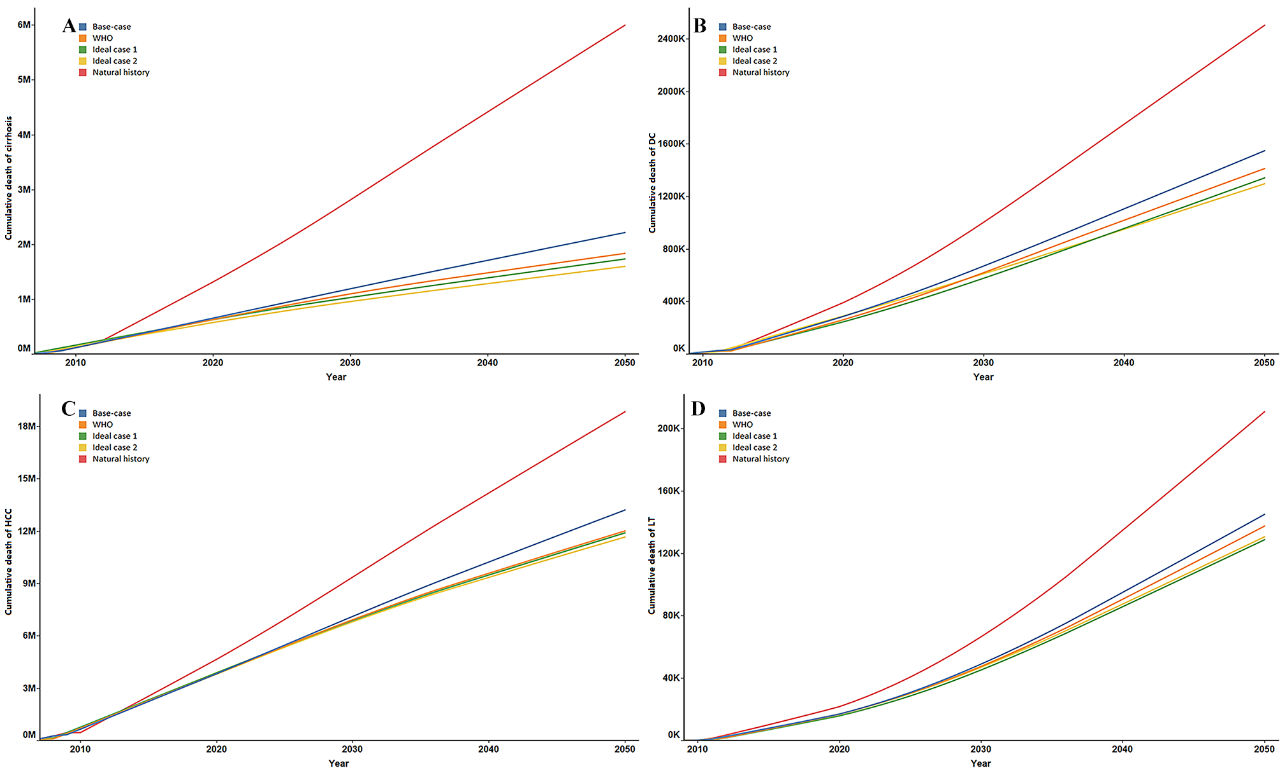


**Supplementary** **Figure 3. Cumulative cirrhosis, DC, HCC, LT death**

A: cirrhosis death; B: decompensated cirrhosis death; C: hepatocellular carcinoma death; D: liver transplantation death


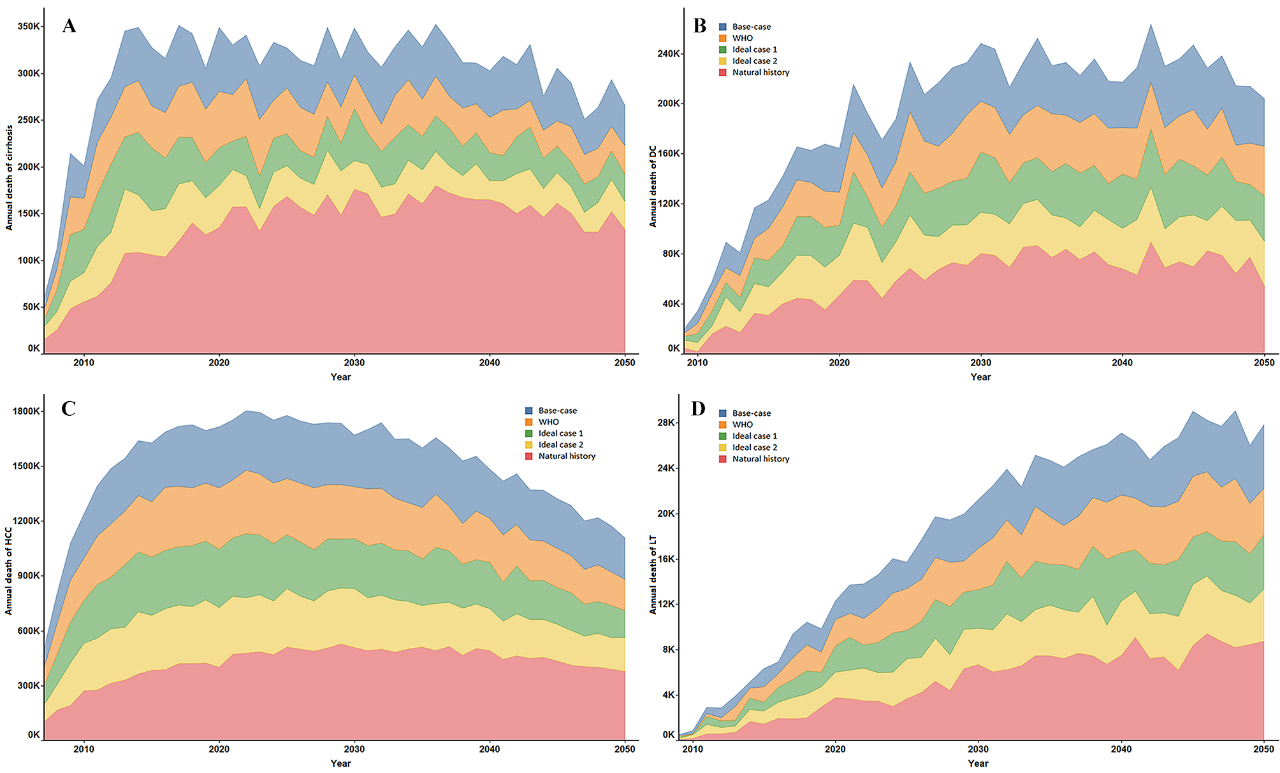


**Supplementary Figure 4. Annual cirrhosis, DC, HCC, LT death number**

A: cirrhosis death; B: decompensated cirrhosis death; C: hepatocellular carcinoma death; D: liver transplantation death
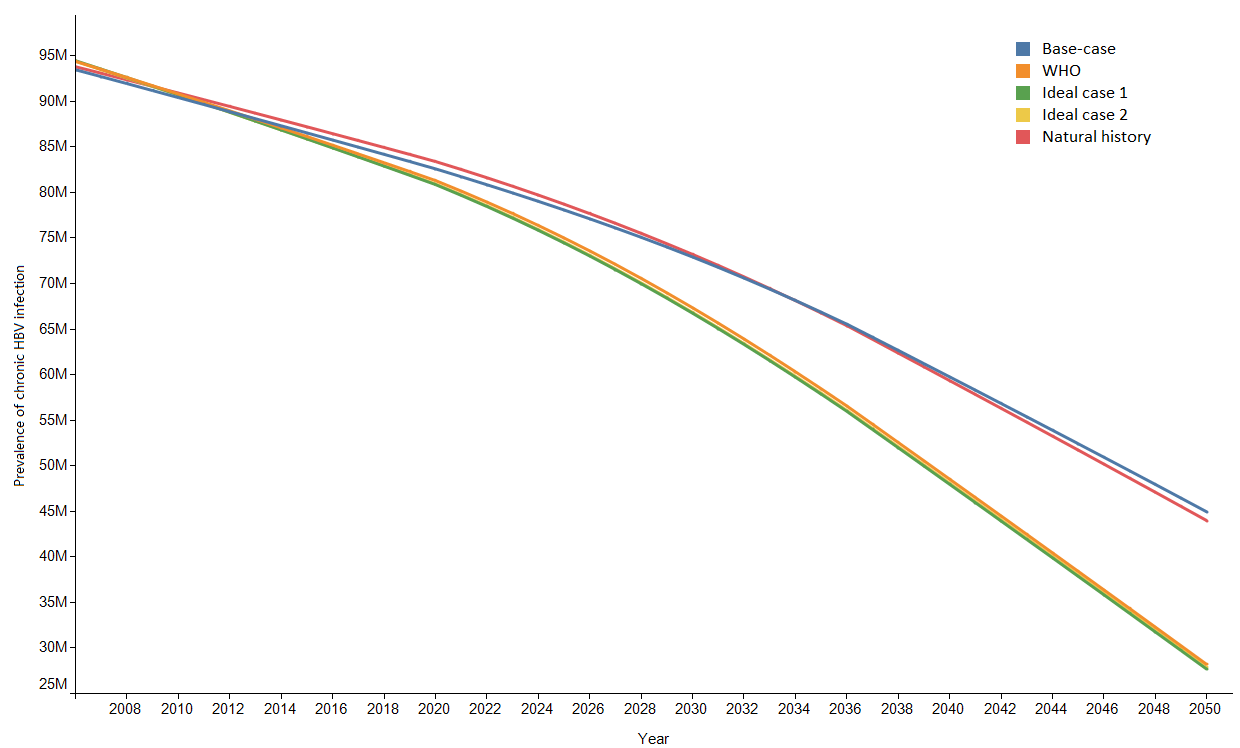


**Supplementary Figure 5. Total chronic HBV infection prevalence**

**Supplementary Table 11. Cumulative and annual incidence number of complications and related-death**

|  | **Natural** | | **Base-case** | | **WHO** | | **Ideal 1** | | **Ideal 2** | | | | |
| --- | --- | --- | --- | --- | --- | --- | --- | --- | --- | --- | --- | --- | --- |
| **year** | **2030** | **2050** | **2030** | **2050** | **2030** | **2050** | **2030** | **2050** | **2030** | | **2050** | | |
| **Cumulative incidence number (million for C, DC, HCC; thousand for LT)** | | | | | | | |  | |  | | |  |
| C | 17.19 | 28.54 | 11.26  (65.5%) | 17.16  (60.14%) | 10.68  (94.91%) | 14.53  (84.64%) | 10.07  (94.3%) | 13.81  (95.03%) | 9.97  (93.35%) | | 13.48  (92.76%) | | |
| DC | 4.19 | 9.23 | 2.67  (63.7%) | 5.66  (61.35%) | 2.48  (93.04%) | 5.00  (88.31%) | 2.35  (94.85%) | 4.76  (95.11%) | 2.40  (96.85%) | | 4.73  (94.55%) | | |
| HCC | 11.42 | 21.47 | 8.57  (75.06%) | 15.05  (70.11%) | 8.41  (98.17%) | 13.52  (89.81%) | 8.27  (98.32%) | 13.27  (98.17%) | 8.28  (98.36%) | | 13.15  (97.23%) | | |
| LT | 138.89 | 320.42 | 99.22  (71.44%) | 213.72  (66.7%) | 96.45  (97.2%) | 197.33  (92.33%) | 89.91  (93.22%) | 183.35  (92.91%) | 94.15  (97.61%) | | 186.96  (94.74%) | | |
| **total** | **32.93** | **59.56** | **22.59** | **38.10** | **21.68** | **33.25** | **20.79** | **32.02** | **20.75** | | **31.54** | | |
| C death | 2.81 | 5.93 | 1.19  (42.3%) | 2.19  (37.01%) | 1.11  (93.62%) | 1.82  (82.72%) | 1.03  (92.75%) | 1.72  (94.47%) | 0.97  (87.26%) | | 1.60  (87.91%) | | |
| DC death | 0.98 | 2.48 | 0.67  (68.65%) | 1.55  (62.54%) | 0.62  (92.26%) | 1.40  (90.23%) | 0.58  (93.09%) | 1.33  (95.12%) | 0.61  (98.39%) | | 1.30  (92.97%) | | |
| HCCdeath | 9.39 | 18.57 | 7.12  (75.78%) | 13.00  (70.01%) | 6.99  (98.25%) | 11.78  (90.58%) | 6.92  (98.9%) | 11.6  (98.49%) | 6.90  (98.67%) | | 11.43  (97.1%) | | |
| LT death | 62.22 | 214.63 | 46.58  (74.86%) | 146.84  (68.41%) | 46.03  (98.83%) | 138.54  (94.35%) | 43.45  (94.39%) | 129.59  (93.54%) | 44.95  (97.65%) | | 131.5  (94.92%) | | |
| **totaldeath** | **13.24** | **27.19** | **9.02** | **16.89** | **8.77** | **15.13** | **8.57** | **14.77** | **8.53** | | **14.46** | | |
|  |  |  | **vs. natural** | | **vs. base** | | **vs. WHO** | | **vs. WHO** | | | | |
| **Annual incidence number (thousand)** | | | | | | |  | |  | | |  |  |
| C | 715.72 | 359.87 | 380.77 | 203.25 | 274.65 | 110.24 | 290.77 | 106.06 | 239.75 | | 96.93 | | |
| DC | 276.53 | 210.27 | 167.24 | 136.24 | 130.65 | 107.11 | 139.29 | 97.89 | 112.16 | | 127.19 | | |
| HCC | 541.24 | 411.44 | 294.78 | 258.01 | 325.84 | 168.84 | 328.10 | 147.27 | 340.01 | | 165.33 | | |
| LT | 7.77 | 8.35 | 6.16 | 5.10 | 6.29 | 4.33 | 5.28 | 4.36 | 4.70 | | 3.32 | | |
| **total** | **1541.26** | **989.93** | **848.95** | **602.6** | **737.43** | **390.52** | **763.44** | **355.58** | **696.62** | | **392.77** | | |
|  |  |  |  |  | **13.14%** | **35.19%** | **10.07%** | **40.99%** | **17.94%** | | **34.82%** | | |
| C death | 176.00 | 133.04 | 49.71 | 42.98 | 36.60 | 30.33 | 55.61 | 29.35 | 30.45 | | 30.02 | | |
| DC death | 80.69 | 54.38 | 46.09 | 38.15 | 40.36 | 39.53 | 48.44 | 36.45 | 32.45 | | 35.50 | | |
| HCCdeath | 507.92 | 377.82 | 280.85 | 226.74 | 283.94 | 168.70 | 272.03 | 150.53 | 290.07 | | 183.36 | | |
| LT death | 6.69 | 8.75 | 4.27 | 5.57 | 3.64 | 4.12 | 3.40 | 4.76 | 3.20 | | 4.61 | | |
| **totaldeath** | **771.3** | **573.99** | **380.92** | **313.44** | **364.54** | **242.68** | **379.48** | **221.09** | **389.23** | | **253.49** | | |
|  |  |  |  |  | **4.30%** | **22.58%** | **0.38%** | **29.46%** | **6.50%** | | **19.13%** | | |
|  |  |  |  |  | **vs. base** | | **vs. base** | | **vs. base** | | | | |

C: cirrhosis

**Reference:**

1. Chinese Center For Disease Control And Prevention. [Serological epidemiologic survey of hepatitis B in China](in Chinese): People's Medical Publishing House, 2010.

2. National Bureau of Statistics of China. Chinese Statistical Yearbook http://www.stats.gov.cn/english/Statisticaldata/AnnualData/. Assessed 10 May 2019.

3. Chinese Center for Disease Control and Prevention. The Data-center of China Public Health Science. 2018. http://www.phsciencedata.cn/Share/en/index.jsp. Assesed 10 May 2019..

4. Fu-Qiang C, Hui Z. Epidemics and control of hepatitis B in China. Chin J Viral Dis. 2018.8(4):257-264.

5. Zhang Q, Qi W, Wang X, Zhang Y, Xu Y, Qin S, et al. Epidemiology of Hepatitis B and Hepatitis C Infections and Benefits of Programs for Hepatitis Prevention in Northeastern China: A Cross-Sectional Study. Clin Infect Dis. 2016, 62(3):305-312. doi: 10.1093/cid/civ859.

6. World Health Organization. Global health sector strategy on viral hepatitis 2016-2021: Publication details. 2016. <https://www.who.int/hepatitis/strategy2016-2021/ghss-hep/en/>. Assessed 10 May 2019.

7. Toy M, Salomon JA, Jiang H, Gui H, Wang H, Wang J, et al. Population health impact and cost-effectiveness of monitoring inactive chronic hepatitis B and treating eligible patients in Shanghai, China. Hepatology. 2014, 60(1):46-55. doi: 10.1002/hep.26934. Epub 2014 May 27.

8. Toy M, Hutton DW, So SK. Cost-Effectiveness and Cost Thresholds of Generic and Brand Drugs in a National Chronic Hepatitis B Treatment Program in China. PLoS One 2015;10:e0139876. doi: 10.1371/journal.pone.0139876.

9. Sharma SK, Saini N, Chwla Y. Hepatitis B virus: inactive carriers. Virol J. 2005, 2:82. doi: 10.1186/1743-422X-2-82.

10. Wang H, Ru GQ, Yan R, Zhou Y, Wang MS, Cheng MJ. Histologic Disease in Chinese Chronic Hepatitis B Patients With Low Viral Loads and Persistently Normal Alanine Aminotransferase Levels. J Clin Gastroenterol. 2016, 50(9):790-796. doi: 10.1097/MCG.0000000000000544.

11. Tseng TC, Liu CJ, Yang HC, Su TH, Wang CC, Chen CL,et al. Serum hepatitis B surface antigen levels help predict disease progression in patients with low hepatitis B virus loads. Hepatology. 2013, 57(2):441-450. doi: 10.1002/hep.26041.

12. Invernizzi F, Vigano M, Grossi G, Lampertico P. The prognosis and management of inactive HBV carriers. Liver Int. 2016;36 Suppl 1:100-104. doi: 10.1111/liv.13006.

13. Chu CM, Liaw YF. HBsAg seroclearance in asymptomatic carriers of high endemic areas: appreciably high rates during a long-term follow-up. Hepatology. 2007, 45(5):1187-1192. doi: 10.1002/hep.21612.

14. Wong WW, Woo G, Heathcote EJ, Krahn M. Disease burden of chronic hepatitis B among immigrants in Canada. Can J Gastroenterol. 2013;27:137-147. doi: 10.1155/2013/924640.

15. Fattovich G, Bortolotti F, Donato F. Natural history of chronic hepatitis B: special emphasis on disease progression and prognostic factors. J Hepatol. 2008;48:335-352. doi: 10.1016/j.jhep.2007.11.011.

16. Simonetti J, Bulkow L, McMahon BJ, Homan C, Snowball M, Negus S, et al. Clearance of hepatitis B surface antigen and risk of hepatocellular carcinoma in a cohort chronically infected with hepatitis B virus. Hepatology. 2010;51:1531-1537. doi: 10.1002/hep.23464.

17. Zhu LG, Tian H, Jiang J, Song C, Zou Y, Xu JF, et al. [Epidemiological characteristics of HBeAg reversion in chronic hepatitis B patients with HBeAg seroconversion in Jiangsu province, 2012-2014]. Zhonghua Liu Xing Bing Xue Za Zhi. 2017;38:43-48. doi: 10.3760/cma.j.issn.0254-6450.2017.01.008.

18. Yang HI, Hung HL, Lee MH, Liu J, Jen CL, Su J, et al. Incidence and determinants of spontaneous seroclearance of hepatitis B e antigen and DNA in patients with chronic hepatitis B. Clin Gastroenterol Hepatol. 2012;10:527-534.e521-522. doi: 10.1016/j.cgh.2011.12.019.

19. Peng CY, Chien RN, Liaw YF. Hepatitis B virus-related decompensated liver cirrhosis: benefits of antiviral therapy. J Hepatol 2012;57:442-450. doi: 10.1016/j.jhep.2012.02.033.

20. Wong GL, Chan HL, Mak CW, Lee SK, Ip ZM, Lam AT, et al. Entecavir treatment reduces hepatic events and deaths in chronic hepatitis B patients with liver cirrhosis. Hepatology. 2013;58:1537-1547. doi: 10.1002/hep.26301.

21. Zeng H, Chen W, Zheng R, Zhang S, Ji JS, Zou X, et al. Changing cancer survival in China during 2003-15: a pooled analysis of 17 population-based cancer registries. Lancet Glob Health. 2018;6:e555-e567. doi: 10.1016/S2214-109X(18)30127-X.

22. Wei Q, Xu X, Wang C, Zhuang R, Zhuang L, Zhou L, et al. Efficacy and Safety of a Steroid-Free Immunosuppressive Regimen after Liver Transplantation for Hepatocellular Carcinoma. Gut Liver. 2016;10:604-610. doi: 10.5009/gnl15017.

23. China Liver Transplant Registry. China Liver Transplant Registry: Transplant Centre. 2019. <http://www.cltr.org/pages/trancenter/trancenter_map.jsp>. Assessed in 10 May 2019.

24. Ming YZ, Zhuang Q, Tu B, et al. Liver Transplantation in China. IntechOpen, 2018. doi: 10.5772/intechopen.81230.

25. Wang SF, Chen XP.[Overview of the development of liver transplantation in China]. Chin J Organ Transplant. 2018, 39(5): 307-310. doi: 10.3760/cma.j.issn.0254-1785.2018.05.011. (in Chinese).

26. Terrault NA, Lok ASF, McMahon BJ, Chang KM, Hwang JP, Jonas MM, et al. Update on prevention, diagnosis, and treatment of chronic hepatitis B: AASLD 2018 hepatitis B guidance. Hepatology. 2018, 67(4):1560-1599. doi: 10.1002/hep.29800.

27. Gish RG, Given BD, Lai CL, Locarnini SA, Lau JY, Lewis DL, et al. Chronic hepatitis B: Virology, natural history, current management and a glimpse at future opportunities. Antiviral Res. 2015;121:47-58. doi: 10.1016/j.antiviral.2015.06.008.

28. Choi J, Kim HJ, Lee J, Cho S, Ko MJ, Lim YS. Risk of Hepatocellular Carcinoma in Patients Treated With Entecavir vs Tenofovir for Chronic Hepatitis B: A Korean Nationwide Cohort Study. JAMA Oncol. 2019;5:30-36. doi: 10.1001/jamaoncol.2018.4070.

29. Kayaaslan B, Guner R. Adverse effects of oral antiviral therapy in chronic hepatitis B. World J Hepatol. 2017;9:227-241. doi: 10.4254/wjh.v9.i5.227.

30. Wei L, Hu S, Hou J, Liu G, Ren H, Duan Z, et al. A Novel Estimation of the Impact of Treatment with Entecavir on Long-Term Mortality, Morbidity, and Health Care Costs of Chronic Hepatitis B in China. Value Health Reg Issues. 2013;2:48-56. doi: 10.1016/j.vhri.2013.02.002.

31. Hontelez JA, Chang AY, Ogbuoji O, de Vlas SJ, Barnighausen T, Atun R. Changing HIV treatment eligibility under health system constraints in sub-Saharan Africa: investment needs, population health gains, and cost-effectiveness. AIDS. 2016;30:2341-2350. doi: 10.1097/QAD.0000000000001190.

32. World Health Organization. Consolidated guidelines on the use of antiretroviral drugs for treating and preventing HIV infection: what’s new. 2015. <https://www.who.int/hiv/pub/arv/policy-brief-arv-2015/en/>. Assessed in 10 May 2019.

33. Cleveland WS. Robust locally weighted regression and smoothing scatterplots. J Am Stat Assoc. 1979;74:829-836.

34. Berger JA, Hautaniemi S, Järvinen A-K, Edgren H, Mitra SK, Astola J. Optimized LOWESS normalization parameter selection for DNA microarray data. BMC bioinformatics. 2004;5:1-13. doi: 10.1186/1471-2105-5-194.

35. Talgorn B, Audet C, Le Digabel S, Kokkolaras M. Locally weighted regression models for surrogate-assisted design optimization. Optim Eng. 2018;19:213-238. doi: https://doi.org/10.1007/s11081-017-9370-5.

36. World Health Organization; International Agency for Research on Cancer. Cancer Incidence in Five Continents Time Trends (CI5plus). 2019. http://ci5.iarc.fr/CI5plus/Default.aspx. Assessed 12 Oct 2019

37. World Health Organization; International Agency for Research on Cancer. Globocan: 2018 China. 2019. http://gco.iarc.fr/today/data/factsheets/populations/160-china-fact-sheets.pdf. Assessed 12 Oct 2019.

38. CDA Foundation. POLARIS observatory: hepatitis B in China. 2019 http://cdafound.org/polaris-hepB-dashboard. Assessed 12 Oct 2019.

39. Institute for Health Metrics and Evaluation. Global Burden Disease 2017 data. 2020. http://ghdx.healthdata.org/gbd-results-tool. Assesed 20 Jun 2020.

40. China Liver Transplant Registry. Statistical Analysis. 2020. http://www.cltr.org/pages/statistics/statistics_livercount.jsp. Assesed 20 Jun 2020.

41. Mokdad AA, Lopez AD, Shahraz S, Lozano R, Mokdad AH, Stanaway J, et al. Liver cirrhosis mortality in 187 countries between 1980 and 2010: a systematic analysis. BMC Med. 2014, 12:145. doi: 10.1186/s12916-014-0145-y.

42. GBD 2017 Cirrhosis Collaborators. The global, regional, and national burden of cirrhosis by cause in 195 countries and territories, 1990-2017: a systematic analysis for the Global Burden of Disease Study 2017. Lancet Gastroenterol Hepatol 2020;5:245-266. doi: 10.1016/S2468-1253(19)30349-8.

43. Chu CM, Liaw YF. HBsAg seroclearance in asymptomatic carriers of high endemic areas: appreciably high rates during a long-term follow-up. Hepatology. 2007, 45(5):1187-1192. doi: 10.1002/hep.21612.
